# Dimensional reduction in networks of non-Markovian spiking neurons: Equivalence of synaptic filtering and heterogeneous propagation delays

**DOI:** 10.1371/journal.pcbi.1007404

**Published:** 2019-10-08

**Authors:** Maurizio Mattia, Matteo Biggio, Andrea Galluzzi, Marco Storace

**Affiliations:** 1 Istituto Superiore di Sanità, Roma, Italy; 2 DITEN, University of Genoa, Genova, Italy; University of Pittsburgh, UNITED STATES

## Abstract

Message passing between components of a distributed physical system is non-instantaneous and contributes to determine the time scales of the emerging collective dynamics. In biological neuron networks this is due in part to local synaptic filtering of exchanged spikes, and in part to the distribution of the axonal transmission delays. How differently these two kinds of communication protocols affect the network dynamics is still an open issue due to the difficulties in dealing with the non-Markovian nature of synaptic transmission. Here, we develop a mean-field dimensional reduction yielding to an effective Markovian dynamics of the population density of the neuronal membrane potential, valid under the hypothesis of small fluctuations of the synaptic current. Within this limit, the resulting theory allows us to prove the formal equivalence between the two transmission mechanisms, holding for any synaptic time scale, integrate-and-fire neuron model, spike emission regimes and for different network states even when the neuron number is finite. The equivalence holds even for larger fluctuations of the synaptic input, if white noise currents are incorporated to model other possible biological features such as ionic channel stochasticity.

## Author summary

Understanding the collective behavior of the intricate web of neurons composing a brain is one of the most challenging and complex tasks of modern neuroscience. Part of this complexity resides in the distributed nature of the interactions between the network components: for instance, the neurons transmit their messages (through spikes) with delays, which are due to different axonal lengths (i.e., communication distances) and/or non-instantaneous synaptic transmission. In developing effective network models, both of these aspects have to be taken into account. In addition, a satisfactory description level must be chosen as a compromise between simplicity and faithfulness in reproducing the system behavior. Here we propose a method to derive an effective theoretical description—validated through network simulations at microscopic level—of the neuron population dynamics in many different working conditions and parameter settings, valid for any synaptic time scale. In doing this we assume relatively small instantaneous fluctuations of the input synaptic current. As a by-product of this theoretical derivation, we prove analytically that a network with non-instantaneous synaptic transmission with fixed spike delivery delay is equivalent to a network characterized by a suited distribution of spike delays and instantaneous synaptic transmission, the latter being easier to treat.

## Introduction

Large distributed systems like brain neuronal networks often have to satisfy both timing and space constraints, irrespective of their size. Indeed, they have to be compact in order to be portable, and at the same time the neurons have to communicate always to the same pace, in part determined by the environment [1]. Communication through spikes makes this possible in a distributed highly-parallel architecture with low power density. Spike communication in these networks relies on both a synaptic low-pass filtering and a suited distribution of axonal propagation delays: a heterogeneous transmission mechanism, which in turn affects the collective dynamics of the network. As a consequence, even in the simplest modeling condition of point-like spiking neurons, the microscopic dynamics of their state variable (i.e., the membrane potential *V*(*t*)) is non-Markovian, and hence multi-dimensional through Markovian embedding [2, 3]. This microscopic dynamics spans a wide variety of time scales, affecting the stability of various network dynamics [4], the selectivity in transmitting information [5] and the reactivity to suddenly appearing exogenous stimuli [6].

Despite the long history of attempts in statistical physics to work out an effective dimensional reduction leading to a Markovian description of the dynamics of a non-Markovian system [2, 7], a theoretical framework valid for any correlation time of the synaptic input and any neuronal activity regime is still missing. Indeed, theoretical approaches including non-instantaneous transmission rely on approximations valid only for relatively small time scales [6, 8–10], or for quasi-adiabatic dynamical regimes [11, 12], or for neurons working with low-noise supra-threshold inputs [13, 14].

Here, we address this issue by developing a theoretical framework in which from small to large synaptic time scales the same dynamical model holds for the instantaneous firing rate of spiking neuron networks. Starting from the population density dynamics of single-neuron state variables under mean-field approximation, we extend to the colored-noise case the spectral expansion of the associated Fokker-Planck (FP) equation previously derived under white-noise assumption [15]. Resorting to a kind of central moment closure method [16], a zero-th order approximation of the population density dynamics is worked out, recovering the same theoretical framework as for the white-noise case. In this framework, the projections of the population density onto the basis composed of the non-stationary eigenmodes of the FP operator (i.e., the eigenfunctions with non-zero eigenvalues) are used to fully represent the state of the network. This basis moves driven by the time-varying moments of the synaptic current which depends on a low-pass filtered version of the network spiking activity. In this way, the basis effectively adapts to the population density evolution and the network dynamics is described by a compact system, valid for a wide class of models and dynamical regimes. We validate the effectiveness of this theoretical description showing a remarkable agreement with microscopic simulations of networks with various integrate-and-fire (IF) neuron models (leaky, exponential and VLSI), spike emission regimes (sub- and supra-threshold) and dynamical states (asynchronous states, corresponding to stable equilibrium points of the instantaneous firing rate and oscillating synchronous states, corresponding to limit cycles), even when finite-size fluctuations are taken into account.

Finally, we prove a formal equivalence between the two transmission mechanisms: a suited distribution of spike transmission delays between neurons allows to fully reproduce the firing rate dynamics of the same network having instead non-instantaneous synaptic transmission. It is then not by chance that both strategies coexist in a huge cellular network like the one composing our brain. Indeed, thanks to such interchangeability, the balance between these mechanisms might be optimally tuned to satisfy both compactness and communication constraints.

## Results

### Theoretical results

#### Full population density model as a set of interacting Markovian systems

We consider a network composed of *N* integrate-and-fire (IF) neurons, each receiving an input current *I*(*t*) resulting from the low-pass filtered linear combination of the spikes emitted at times *t*_*j*_, with Poissonian distribution, by a subset of presynaptic cells in the network [9, 17]. For simplicity, here we assume a first-order dynamics for synaptic current *I*, with decay time *τ*_*s*_ and constant efficacy $J:\tau_{s}\;\dot{I}=-I+\tau_{m}\;J\;\sum_{j}\delta \left(t-t_{j}\right)/R$. The membrane potential *V*(*t*) of an IF neuron evolves according to the general equation $\tau_{m}\;\dot{V}=-f\left(V\right)+R\;\left(I+I_{ext}\right)$, with membrane decay constant *τ*_m_, neuron resistance *R* = *τ*_m_/*C*_m_, membrane capacitance *C*_*m*_ and a voltage-dependent leakage drift *f*(*V*), which depends on the model neuron type. For instance, *f*(*V*) is a constant drift for the perfect IF (PIF) neuron and *f*(*V*) = *V* for the leaky IF (LIF) [18, 19].

**Fig 1:**
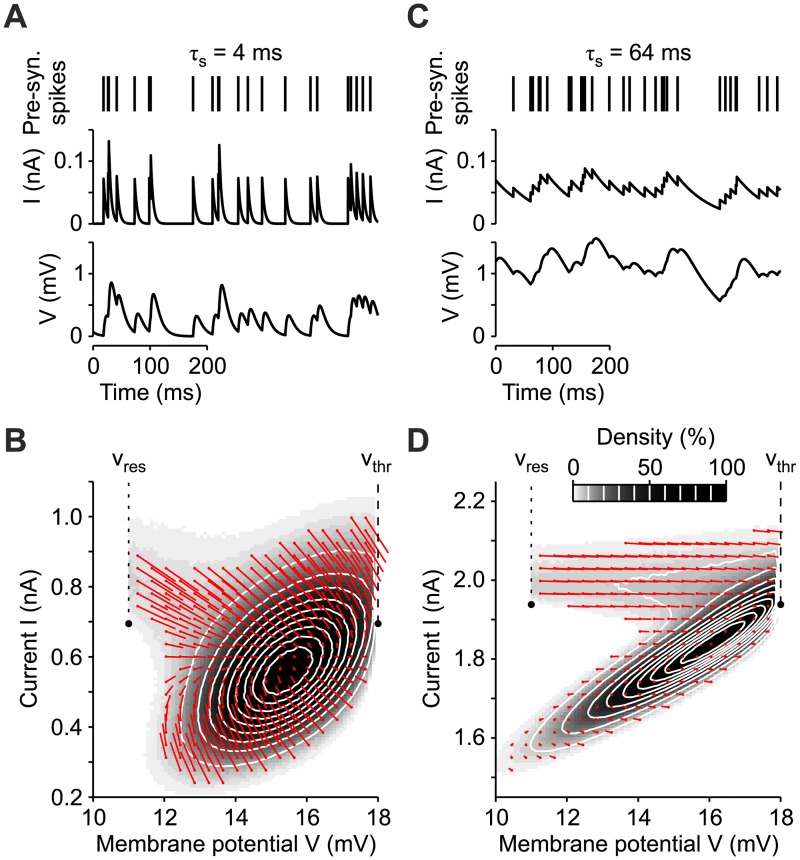
Spike input filtering by synaptic transmission. (A, C) Presynaptic activity modeled by a Poissonian spike train (top) is low pass-filtered by the synaptic transmission to produce the input current *I*(*t*) (middle) to the membrane potential *V*(*t*) (bottom) of a leaky integrate-and-fire (LIF) neuron. Examples are shown for fast (A, *τ*_*s*_ = 4 ms) and slow (C, *τ*_*s*_ = 64 ms) synaptic filtering. (B, D) Stationary distribution in the plane (*V*, *I*) sampled from simulations of the same LIF neurons as in A and C with fast (B) and slow (D) synaptic transmission. Red arrows: mean fluxes of realizations (probability currents) at different (*V*, *I*). *v*_thr_: absorbing barrier representing the spike emission threshold. *v*_res_: reset membrane potential following the emission of a spike. Here *I*_*ext*_ = 0.

Neurons may receive an additional current *I*_*ext*_ modeling external sources, like incoming synaptic input from other networks. Once *V* crosses a threshold value *v*_thr_, a spike is emitted and potential *V* is reset to *v*_res_ < *v*_thr_ (boundary conditions). Fig 1A shows a Poisson spike train (top panel) and the corresponding input current *I*(*t*) (middle panel) and membrane potential *V*(*t*) (bottom panel) for a LIF neuron with *τ*_*s*_ = 4 ms, *τ*_m_ = 10 ms and *I*_*ext*_ = 0.

Due to the boundary conditions, even under stationary regimes single-neuron dynamics is not analytically tractable. This is particularly apparent looking at the stationary probability distribution computed from the long time series partially shown in Fig 1A. The probability distribution of states (*V*, *I*) worked out numerically is shown in Fig 1B. The absorbing barrier in *v*_thr_ and the reentering flux of realizations in *v*_res_ modeling the emission of spikes make this distribution asymmetric [6, 8, 20] and correlation between *V* and *I* non-monotonic, a feature even more apparent for slower synaptic filtering, as shown in Fig 1C and 1D, obtained by increasing *τ*_*s*_ to 64 ms.

Under diffusion approximation, holding for large rate of incoming spikes each only mildly affecting *V* [18, 19], membrane potential dynamics is described by the following system of Langevin equations [2]

$$\begin{array}{c} \tau_{m}\;\dot{V}=-f\left(V\right)+R\left(I+I_{ext}\right)\;,\;\tau_{s}\;\dot{I}=-I+\frac{\mu_{I}+\sigma_{I}\;\xi }{R}\;, \end{array}$$ (1)

where *RI*_*ext*_ = *μ*_*ext*_ + *σ*_*ext*_ *ξ*_*ext*_ is a Gaussian white noise. We remark that the filtering effect of synaptic dynamics makes the noise colored, thus leading to finite correlation time constants. Moreover, *V*(*t*), individually, is not Markovian but *V*(*t*) and *I*(*t*), together, constitute a bi-variate Markov process [2].

**Fig 2:**
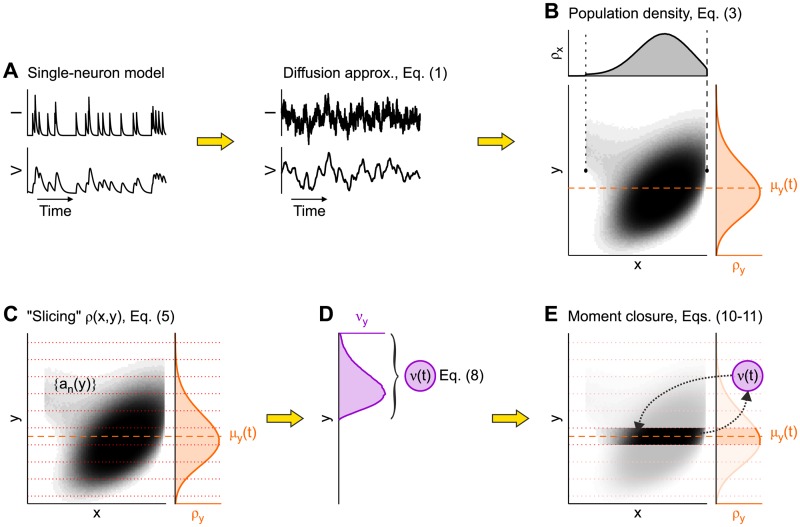
Sketch of the main steps of the theoretical derivation. (A) Diffusion approximation (right) of the single-neuron dynamics. (B) Probability density *ρ*(*x*, *y*) at fixed *t*, with the associated marginal distributions for both membrane potential (*ρ*_*x*_, gray) and current (*ρ*_*y*_, orange). Orange dashed line: instantaneous mean *μ*_*y*_ of the current (see main text for details). (C) “Slices” of the two-dimensional *ρ*(*x*, *y*) bounded by red dotted lines; each “slice” (which actually has infinitesimal thickness) corresponds to a one-dimensional subpopulation of neurons with similar *y* and is described by the expansion coefficients {*a*_*n*_(*y*)} of a suited one-dimensional FP operator. (D) Partial firing rates *ν*_*y*_ (purple) are integrated over *y* to obtain the *ν*(*t*) of the whole network. (E) The instantaneous firing rate *ν*(*t*) is now the only state variable needed to be fed back in order to operate the moment closure and take into account only the “slice” centered around *μ*_*y*_ (the 0-th order approximation).

Fig 2 provides an at-a-glance description of our method to obtain the final population dynamics and is used as a main reference throughout this section. The first intermediate Langevin model (1) corresponds to Fig 2A.

When the mean driving force alone (i.e., the deterministic part of the input *I* + *I*_*ext*_) is not enough to make the membrane potential *V* cross the threshold *v*_*thr*_, the neurons are evolving in a *noise-dominated* (or subthreshold) *regime* and irregular firing occurs, due to the stochastic part of the input. In the opposite case, the emission of an action potential can occur also in the absence of noisy afferent currents, and the neurons are in a *drift-dominated* (or suprathreshold) *regime* of activity, characterized by the regular emission of spike trains [21–23].

The stochastic differential Eq (1) describe a single neuron where the time course of *V*(*t*) and *I*(*t*) is probabilistic. Therefore, the dynamics of *V* and *I* is fully described by the probability density *ρ*(*V*, *I*, *t*). Fig 1B and 1D are examples of distributions of (*V*, *I*) samples obtained from the numerical integration of the single-neuron dynamics under stationary conditions and before assuming the diffusion approximation. The probability density *ρ*(*V*, *I*, *t*) describes (under diffusion approximation) how this distribution evolves in time.

If we consider a network of statistically identical neurons, each with a different realization of the stochastic input, *ρ*(*V*, *I*, *t*) can be considered a population density. The population density approach [2, 24] provides a method to analyze the dynamics of ensembles of connected neurons, relating the firing properties of a network to the features of single neurons and of their synaptic connections. This is done by assuming the “mean-field” hypothesis, which allows to use the probability density for independent neurons in a “self-consistent” way. According to this theoretical ansatz, the infinitesimal moments of *RI*

$$\begin{array}{c} \begin{array}{c} \mu_{I}\left(t\right)=\tau_{m}\;J\;C\;\nu \left(t\right)\\ \sigma_{I}^{2}\left(t\right)=\tau_{m}\;J^{2}\;C\;\nu \left(t\right) \end{array} \end{array}$$ (2)

characterize the white noise *ξ* driving the activity-dependent synaptic current [8, 25] as a function of the network firing rate *ν*(*t*) (i.e., the average number of spikes each neuron emits per time unit) and the average number of presynaptic contacts *C* (≤ *N*) per neuron. The probability density *ρ*(*x*, *y*, *t*) to find neurons at time *t* with membrane potential *V*(*t*) = *x* and synaptic current *I*(*t*) = *y*/*R* follows the FP equation [2] (see Fig 2B):

$$\begin{array}{c} \begin{array}{ccc} \partial_{t}\rho & = & -\partial_{x}F_{x}\left(x,y\right)-\partial_{y}F_{y}\left(y,\nu \right)\\ & \equiv & \frac{1}{\tau_{m}}\;\partial_{x}[(f\left(x\right)-y-\mu_{ext})+\frac{1}{2}\sigma_{ext}^{2}\partial_{x}]\rho +\\ & & \frac{1}{\tau_{s}}\;\partial_{y}[\left(y-\mu_{I}\right)+\frac{1}{2}\sigma_{I}^{2}\partial_{y}]\rho \\ & \equiv & (L_{x}+L_{y})\rho \;. \end{array} \end{array}$$ (3)

This is a continuity equation in which the density changes are determined by the divergence of the probability current $\left(F_{x},F_{y}\right)$ (i.e., the flux of realizations) together with few specific boundary conditions (see Fig 1): i) spike emission as an absorbing barrier at *x* = *v*_thr_ (only for *y* > 0 if *σ*_*ext*_ = 0), ii) reentering flux of absorbed realizations at the reset potential *v*_res_ and iii) lower bound for the membrane potential implemented as a reflecting barrier at *v*_*min*_ ≤ *v*_*res*_ [8, 15, 26, 27].

In general, Eq (3) is hard to solve and only approximated solutions (mainly under stationary conditions) have been worked out [2, 6, 7, 28]. Here we derive a solution for this two-dimensional problem without making any assumption like stationarity in time and restricted ranges for the synaptic filtering timescale. We extend the spectral expansion approach exploited to study the one-dimensional case [15, 26], by describing *ρ*(*x*, *y*, *t*) as the superposition of an *infinite set of one-dimensional interacting sub-populations*; each sub-population collects the neurons that at time *t* receive a synaptic current *y*/*R*. Therefore we have *iso-current sub-populations* labeled by *y* and changing in time as the realizations move along the *y*-dimension due to the action of $F_{y}$. In other words, we “slice” the two-dimensional diagrams of the probability current $\left(F_{x},F_{y}\right)$ corresponding to *ρ*(*x*, *y*, *t*) along the *y* direction, thus obtaining probability densities $\rho (x,y,t){|}_{y=\bar{y}}$, as sketched in Fig 2C.

The dynamics of each iso-current sub-population is studied, as in [15], by projecting *ρ* at fixed *y* on the eigenfunctions {|*ϕ*_*m*_〉} of a suited one-dimensional FP operator, which here we set to be

$$\begin{array}{c} L_{xy}=\frac{1}{\tau_{m}}\partial_{x}\left[f\left(x\right)-y-\mu_{ext}\right]+\frac{1}{2\tau_{m}}[\sigma_{ext}^{2}+\sigma^{2}\left(y\right)]\partial_{x}^{2}, \end{array}$$ (4)

with an additional diffusion term *σ*^2^(*y*) with respect to $L_{x}$ of Eq (3), which is the variance of an *arbitrary* Gaussian white noise depending on the state variable *y*.

As shown later, this additional term will play a crucial role in our dimensional reduction. Indeed, *σ*^2^(*y*) will be chosen self-consistently to take into account the statistical variability of the input currents received by all the iso-current subpopulations. This unconventional choice allows us to use an expansion in eigenfunctions of this FP operator, with coefficients $a_{n}\left(y,t\right)=\langle \psi_{n}|\rho \rangle =\int_{v_{\text{min}}}^{v_{\text{thr}}}\psi_{n}\left(x,y\right)\;\rho \left(x,y,t\right)\;dx$, which now explicitly depend on the term *y* and where 〈*ψ*_*n*_| is the *n*-th eigenfunction of the adjoint operator of $L_{xy}$. The stationary mode with eigenvalue λ_0_ = 0 has 〈*ψ*_0_| = 1, thus *a*_0_(*y*, *t*) is the marginal probability density $\rho_{y}\left(y,t\right)=\int_{v_{\text{min}}}^{v_{\text{thr}}}\rho \left(x,y,t\right)\;dx$ (Fig 2, orange distribution).

The dynamics of *a*_*n*_ can be worked out by deriving *a*_*n*_ with respect to time [15, 26], and rewriting $L_{x}=L_{xy}-\frac{1}{2\tau_{m}}\sigma^{2}\left(y\right)\partial_{x}^{2}$:

$$\begin{array}{c} \begin{array}{ccc} {\dot{a}}_{n} & = & \left\langle \psi_{n}|\left(L_{x}+L_{y}\right)\rho \right\rangle =\left\langle \psi_{n}|L_{x}\rho \right\rangle +\left\langle \psi_{n}|L_{y}\rho \right\rangle =\\ & = & \left\langle \psi_{n}|L_{xy}\rho \right\rangle -\frac{\sigma^{2}\left(y\right)}{2\tau_{m}}\left\langle \psi_{n}|\partial_{x}^{2}\rho \right\rangle +\left\langle \psi_{n}|L_{y}\rho \right\rangle =\\ & = & \lambda_{n}a_{n}-\frac{\sigma^{2}\left(y\right)}{2\tau_{m}}\sum_{q}a_{q}\left\langle \psi_{n}|\partial_{x}^{2}\phi_{q}\right\rangle +\sum_{q}\left\langle \psi_{n}|L_{y}\;a_{q}\phi_{q}\right\rangle \;. \end{array} \end{array}$$ (5)

where |*ϕ*_*q*_〉 and 〈*ψ*_*n*_| are the eigenfunctions of the operator $L_{xy}$ and of its adjoint, respectively. For *n* = 0, the dynamics of *ρ*_*y*_(*y*, *t*) = *a*_0_(*y*, *t*) can be worked out, as both $L_{y}$ and {*a*_*q*_} do not depend on *x*:

$$\begin{array}{c} {\dot{\rho }}_{y}=L_{y}\;\rho_{y}-\rho_{y}\frac{\sigma^{2}\left(y\right)}{2\tau_{m}}\int_{v_{\text{min}}}^{v_{\text{thr}}}\partial_{x}^{2}\phi_{0}\left(x,y\right)\;dx\;. \end{array}$$

The integral on the r.h.s. can be solved by parts and turns out to be 0 thanks to both the conservation of the flux of realizations exiting from *v*_thr_ and re-entering in *v*_res_ ($\partial_{x}\phi_{0}{|}_{x=v_{\text{thr}}}=\partial_{x}\phi_{0}{{|}_{x={v_{\text{res}}}^{+}}-\partial_{x}\phi_{0}|}_{x={v_{\text{res}}}^{-}}$) and the reflecting barrier condition in *v*_min_, provided that the reasonable assumption *ϕ*_0_(*v*_min_, *y*) = 0 holds (this is surely true for *v*_min_ → −∞, as *ϕ*_0_ is a probability density). Thus, the *ρ*_*y*_ dynamics reduces to

$$\begin{array}{c} {\dot{\rho }}_{y}=L_{y}\;\rho_{y}=\partial_{y}\frac{\left(y-\mu_{I}\right)\rho_{y}}{\tau_{s}}+\frac{\sigma_{I}^{2}}{2\tau_{s}}\;\partial_{y}^{2}\rho_{y}\;. \end{array}$$ (6)

Note that this expression can be derived directly from the FP Eq (3).

Relying on the same spectral expansion as in [15], each ‘partial’ rate $\nu_{y}\left(y,t\right)=F_{x}\left(v_{\text{thr}},y\right)$ (Fig 2, purple distribution) can be decomposed as follows:

$$\begin{array}{c} \nu_{y}\left(y,t\right)=\rho_{y}\left(y,t\right)\;\Phi \left(y,\nu \right)+\sum_{n\neq 0}a_{n}\left(y,t\right)\;f_{n}\left(y,\nu \right)\;, \end{array}$$ (7)

where the stationary mode contribution is separated from the others. The network firing rate *ν*(*t*) is obtained by integrating the partial rates *ν*_*y*_(*y*, *t*) over *y*, as sketched in Fig 2D:

$$\begin{array}{c} \nu \left(t\right)=\int_{-\infty }^{\infty }F_{x}\left(v_{\text{thr}},y\right)dy\equiv \int_{-\infty }^{\infty }\nu_{y}\left(y,t\right)dy\;, \end{array}$$ (8)

In this framework, $\Phi \left(y,\nu \right)\equiv F_{x}\left(v_{\text{thr}},y\right){|}_{\rho =\phi_{0}}$ is the firing rate of the stationary one-dimensional density *ϕ*_0_ at fixed *y*, while the other nonstationary modes contribute with the fluxes $f_{n}\left(y,\nu \right)\equiv F_{x}\left(v_{\text{thr}},y\right){|}_{\rho =\phi_{n}}$. Both Φ and *f*_*n*_ depend on the total firing rate *ν*(*t*) through the input current moments *μ*_*I*_ and *σ*_*I*_. The main advantage of having transformed the original two-dimensional description into a set of infinite one-dimensional FP equations is that the dynamics described by these equations for each iso-frequency population is known to be tractable for a wide range of network settings. This is crucial to derive an approximated dynamics of *ν*(*t*) valid for any synaptic time scale *τ*_s_, neuron model and dynamical regime, as shown in the following.

#### Dimensional reduction of the firing rate dynamics

Eqs (7) and (8) together are a reformulation of the original problem (3), not a solution. However, in this framework a perturbative approach can be envisaged. From Eq (6), synaptic current *y* results to have time-dependent mean *μ*_*y*_(*t*) = 〈*y*〉 and variance $\sigma_{y}^{2}\left(t\right)=\langle {\left[y-\mu_{y}\left(t\right)\right]}^{2}\rangle$

$$\begin{array}{c} \tau_{s}\;{\dot{\mu }}_{y}=-\mu_{y}+\mu_{I}\;,\;\tau_{s}\;{\dot{\sigma }}_{y}^{2}=-2\sigma_{y}^{2}+\sigma_{I}^{2}\;, \end{array}$$ (9)

like an Ornstein-Uhlenbeck process with nonstationary input moments *μ*_*I*_(*t*) and *σ*_*I*_(*t*) [2]. We remark that $\sigma_{y}^{2}$ should not be confused with the arbitrary and additional diffusion term *σ*^2^(*y*) introduced in the definition of $L_{xy}$: $\sigma_{y}^{2}$ is the variance of the synaptic current *y*, whereas *σ*^2^(*y*) is the state-dependent variance of an unknown diffusion process included in $L_{xy}$, which will be chosen later to obtain a self-consistent equation for the firing rate *ν*(*t*).

Synaptic current displacement in time is $|y-\mu_{y}|=O\left(\sigma_{I}\right)$ and, for small *σ*_*I*_,—i.e., for *σ*_*I*_ ≪ *v*_thr_ – we can assume a negligible role for a *y* distant enough from *μ*_*y*_. This assumption of a narrow *ρ*_*y*_ centered around *y* = *μ*_*y*_ allows to simplify the integrals across the *y* domain weighted by the expansion coefficients {*a*_*n*_(*y*, *t*)}. Indeed, approximations of these integrals can be obtained by expanding in Taylor’s series the *y*-dependent functions in the integrands, as detailed in the S1 Appendix and sketched in Fig 2E.

The next step is to find a self-consistent approximation (neglecting terms of order $O(\sigma_{I})$) of *ν*(*t*) in terms of 0-th order functions Φ_0_(*ν*) ≡ Φ(*μ*_*y*_, *ν*) and *f*_0*n*_(*ν*) ≡ *f*_*n*_(*μ*_*y*_, *ν*) and of corresponding coefficients:

$$\begin{array}{c} \begin{array}{ccc} \nu & \approx & \Phi_{0}\left(\nu \right)+\sum_{n\neq 0}a_{0n}\;f_{0n}\left(\nu \right). \end{array} \end{array}$$ (10)

The dynamics of the *n*-th expansion coefficient is ruled by the following equation, neglecting again terms of order $O(\sigma_{I})$:

$$\begin{array}{c} {\dot{a}}_{0n}\approx \lambda_{0n}\;a_{0n}+{\dot{\mu }}_{y}\sum_{q}\left\langle \partial_{y}\psi_{n}|\phi_{q}\right\rangle {|}_{y=\mu_{y}}a_{0q}, \end{array}$$ (11)

where λ_0*n*_ ≡ λ_*n*_(*μ*_*y*_) (see details in the S1 Appendix).

This is a dimensional reduction of Eq (5) as the coefficients $a_{0n}\left(t\right)\equiv \int_{-\infty }^{\infty }a_{n}\left(y,t\right)\;dy$ do not depend on *y*, and it holds provided that *y* has a narrow distribution across neurons at each time *t* (i.e., small *σ*_*I*_(*t*)), leaving *τ*_*s*_ unconstrained.

To obtain a self-consistent equation for the firing rate *ν*(*t*), we still need to choose a suited *σ*(*y*) in $L_{xy}$. To this purpose, the case of instantaneous synaptic transmission *τ*_*s*_ = 0 can be used as reference, as Eq (3) reduces to a one-dimensional FP equation with the only operator $L_{x0}=-1/\tau_{m}\;\partial_{x}[\mu_{I}+\mu_{ext}-f\left(x\right)]+(\sigma_{ext}^{2}+\sigma_{I}^{2})/\left(2\tau_{m}\right)\partial_{x}^{2}$ [15, 21, 27]. Hence, $L_{xy}{|}_{y=\mu_{y}}$ must tend to $L_{x0}$ for vanishing *τ*_*s*_, which is the case if *σ*^2^(*y*) = *Jy*. Indeed, in this limit $\sigma^{2}\left(\mu_{y}\right)=\tau_{m}\;J^{2}\;C\;\nu =\sigma_{I}^{2}$, being *μ*_*y*_ = *μ*_*I*_ = *τ*_m_ *J C ν* from Eq (9). This implies that eigenvalues λ_0*n*_ and eigenfunctions in Eq (11) are the same as in the one-dimensional case with *δ*-correlated synaptic input, but with collective firing rate *ν*(*t*) = *μ*_*y*_(*t*)/(*τ*_m_ *JC*).

To summarize, $\sigma_{I}^{2}$ is the variance of the white noise driving the synaptic current and depends on *ν*(*t*) (see Eq (2)); $\sigma_{ext}^{2}$ is the variance of the Gaussian white noise representing the external input (see Eq (1)); $\sigma_{y}^{2}$ is the variance of the synaptic current *y* (see Eq (9)); *σ*^2^(*y*) = *Jy* is the variance of the diffusion process appearing in $L_{xy}$ (see Eq (4)), which is different for each iso-current “slice” being dependent on *y*. However, in the limit *τ*_s_ → 0 (where the decay time *τ*_s_ is the relaxation time of *μ*_*y*_(*t*), see Eq (9)), the synaptic transmission becomes instantaneous and $\sigma_{y}^{2}\to \sigma_{I}^{2}/2$, differently from the variance *σ*^2^(*y*) which in the relevant iso-current “slice” at *y* = *μ*_*y*_ is $\sigma_{I}^{2}$.

In conclusion, this dimensional reduction extends the firing rate equation previously worked out for *τ*_*s*_ = 0 [15], and, by combining Eqs (9)–(11) and neglecting $O(\sigma_{I})$ terms, it results to be

$$\begin{array}{c} \left\{ \begin{array}{c} {\dot{\vec{a}}}_{0}=(\Lambda_{0}+W_{0}\;{\dot{\mu }}_{y})\;{\vec{a}}_{0}+{\vec{w}}_{0}\;{\dot{\mu }}_{y}\\ {\dot{\mu }}_{y}=(\mu_{I}\left(\nu \right)-\mu_{y})/\tau_{s}\\ \nu =\Phi_{0}+{\vec{f}}_{0}\cdot {\vec{a}}_{0} \end{array}\;.\right. \end{array}$$ (12)

Here, the infinite vectors ${\vec{a}}_{0}=\left\{a_{0n}\right\}$ and ${\vec{f}}_{0}\left(\nu \right)=\left\{f_{0n}\left(\nu \right)\right\}$ are introduced together with the eigenvalue matrix **Λ**_0_ = diag(λ_0*n*_). Separating stationary from non-stationary modes, the synaptic coupling vector ${\vec{w}}_{0}=\left\{\langle \partial_{y}\psi_{n}|\phi_{0}\rangle {|}_{y=\mu_{y}}\right\}$ and matrix ${\text{W}}_{0}={\left\{\langle \partial_{y}\psi_{n}|\phi_{m}\rangle {|}_{y=\mu_{y}}\right\}}_{m\neq 0}$ are also included, respectively. For the chosen *σ*(*y*), we remark that all these terms depend on the total firing rate *ν*(*t*) only through *μ*_*y*_(*t*).

As in [15], Eq (12) expresses the firing rate *ν*(*t*) as a superposition of contributions due to both the stationary mode for a given *μ*_*y*_(*t*) (i.e., the input-output gain function Φ_0_), and the firing rate due to non-stationary modes (${\vec{f}}_{0}\cdot {\vec{a}}_{0}$) depicting how far the actual density is from the equilibrium. As a result, the farther the system from the equilibrium, the larger the contribution to *ν*(*t*) due to the non-stationary modes. Indeed, under these conditions the expansion coefficients ${\vec{a}}_{0}$ are significantly different from 0, whereas under stationary conditions ${\vec{a}}_{0}=0$ and *ν* = Φ_0_.

The first row of Eq (12) is an infinite set of equations for the infinite expansion coefficients {*a*_0*n*_}. It describes their relaxation dynamics towards an equilibrium point which can change with time, as the stationary one-dimensional density *ϕ*_0_ depends on *μ*_*y*_(*t*). Time scales of this relaxation are dictated by the eigenvalues **Λ**_0_ and the coupling coefficients **W**_0_, the latter being 0 as the synaptic efficacy *J* vanishes. Also Φ_0_, ${\vec{f}}_{0}$, **Λ**_0_ and **W**_0_ depend on *μ*_*y*_(*t*) and on the other changes in the input current received by the neurons. This means that Eq (12) incorporates both slow and fast time scales. The former are due to the slowest *a*_0*n*_ relaxation or to the synaptic low-pass filtering for large *τ*_s_, as pointed out by the second row of Eq (12). On the other hand, fast time scales can be due to the fast changes of the input affecting directly the aforementioned equation coefficients, such as the gain function Φ_0_. This multiscale nature of Eq (12) makes this theoretical representation of general applicability, as shown later.

Eq (12) is one of our main results, obtained following a procedure that can be summarized as follows (see Fig 2). We described the single-neuron dynamics as a two-dimensional Langevin Eq (1), relying on the diffusion approximation. From this, an extended mean-field approximation [25] led us to derive Eq (3), a two-dimensional continuity equation for the dynamics of the population density *ρ*(*x*, *y*, *t*). We then represented *ρ* as the time evolution of its projections onto the axes defined by the eigenfunctions of the FP operator, as shown in Eqs (7) and (8) – an approach similar to the Hartree-Fock method in quantum mechanics but with the main difference that here we take into account also the temporal dimension. Finally, *ρ*(*x*, *y*, *t*) is approximated assuming *y* ≃ *μ*_*y*_(*t*), which is a low-pass filtered version of *μ*_*I*_(*t*). This allows us to operate a central moment closure focusing on the instantaneous firing rate *ν*(*t*). We remark that this dimensional reduction aims at finding an effective model of the firing rate *ν*(*t*), not of the full probability density *ρ*(*x*, *y*, *t*). This means we are not expecting to recover accurately the full dynamics of the neuronal membrane potential *V*(*t*), as detailed later.

#### Equivalence of non-instantaneous synaptic transmission and distribution of axonal delays

So far, we considered the filtering activity operated by local synapses transmitting incoming spikes as a post-synaptic potential non-instantaneous in time. Here, we recall the known dynamics of a network of spiking neurons where synaptic transmission is instantaneous but a distribution of axonal delays is taken into account [15, 27, 29]. In this one-dimensional case (*τ*_*s*_ = 0), the network dynamics can be simply worked out by replacing *ν* in the expressions of *μ*_*I*_ and $\sigma_{I}^{2}$ (see Eq (2)) with the instantaneous rate $\tilde{\nu }\left(t\right)=\int_{0}^{\infty }\nu \left(t-\delta \right)\rho_{d}\left(\delta \right)d\delta$ of spikes received by neurons when a distribution *ρ*_*d*_(*δ*) of axonal transmission delays *δ* is taken into account. Thus, the population density *ρ*_*x*_(*x*, *t*) follows the one-dimensional FP equation

$$\begin{array}{c} \left\{ \begin{array}{c} \partial_{t}\rho_{x}=\frac{1}{\tau_{m}}\;\partial_{x}[(f\left(x\right)-\mu_{I}\left(\tilde{\nu }\right)-\mu_{ext})+\frac{1}{2}(\sigma_{ext}^{2}+\sigma_{I}^{2}\left(\tilde{\nu }\right))\partial_{x}]\rho_{x}\\ \dot{\tilde{\nu }}=(\nu -\tilde{\nu })/\tau_{d} \end{array}\;,\right. \end{array}$$ (13)

with instantaneous firing rate $\nu =\left(\sigma_{ext}^{2}+\sigma_{I}^{2}\right)/\left(2\tau_{m}\right)\partial_{x}\rho_{x}{|}_{x=v_{\text{thr}}}$.

The spectral expansion of this continuity equation leads to a known firing rate equation with coefficients resulting to have a straightforward relationship with those in Eq (12). Indeed, the elements of the synaptic coupling vector and matrix now are $\langle \partial_{\tilde{\nu }}\psi_{n}|\phi_{m}\rangle =\tau_{m}\;J\;C\langle \partial_{y}\psi_{n}|\phi_{m}\rangle {|}_{y=\mu_{y}}$ for any *n* and *m*, since

$$\begin{array}{c} \tilde{\nu }=\frac{\mu_{y}}{\tau_{m}\;J\;C} \end{array}$$

for what shown above. Due to this, the searched firing rate equation equivalent to the 1D FP Eq (13) reduces to

$$\begin{array}{c} \left\{ \begin{array}{c} \dot{\vec{a}}=(\Lambda_{0}+W_{0}\;\tau_{m}\;J\;C\;\dot{\tilde{\nu }})\;\vec{a}+{\vec{w}}_{0}\;\tau_{m}\;J\;C\;\dot{\tilde{\nu }}\\ \dot{\tilde{\nu }}=(\nu -\tilde{\nu })/\tau_{d}\\ \nu =\Phi_{0}+{\vec{f}}_{0}\cdot \vec{a} \end{array}\;,\right. \end{array}$$ (14)

where the specific delay distribution

$$\begin{array}{c} \rho_{d}\left(\delta \right)=\frac{1}{\tau_{d}}e^{-\delta /\tau_{d}}\;\Theta \left(\delta \right) \end{array}$$ (15)

has been taken into account. This leads $\tilde{\nu }$ to be a version of the collective firing rate *ν* smoothed in time by a first-order low-pass filter with decay time *τ*_d_. Θ(*δ*) is the Heaviside function, as only positive transmission delays are admitted.

A comparison between Eqs (12) and (14) remarkably points out the equivalence of having, in a spiking neuron network, a non-instantaneous synaptic transmission or a suited distribution of axonal delays. In Eq (14) the expansion coefficients in $\vec{a}$ refer to the one-dimensional density *ρ*_*x*_(*x*, *t*), while in Eq (12) ${\vec{a}}_{0}$ is only an effective representation of the two-dimensional density *ρ*(*x*, *y*, *t*). Nevertheless, provided that *τ*_s_ = *τ*_d_, both the dynamics seen from the perspective of *ν*(*t*) are the same, as the functions Φ_0_, ${\vec{f}}_{0}$, ${\vec{w}}_{0}$, **W**_0_ and **Λ**_0_ are the same. In other words, under mean-field approximation and for not too large synaptic current fluctuations *σ*_*I*_, having a local synaptic filtering with cut-off frequency 1/*τ*_*s*_ is equivalent to having random axonal delays with exponential distribution (15) and decay constant *τ*_d_ = *τ*_s_. Although such equivalence might appear as not surprising, to the best of our knowledge this is the first proof of its general validity, i.e., holding for any IF neuron model, dynamical regime and synaptic timescale.

### Numerical validation of the theory

The theory (including the equivalence between Eqs (12) and (14)) developed in the previous section has been tested through extensive numerical simulations by using NEST [30] and the high-performance custom simulator implementing the event-based approach described in [31]. The parameters for the numerical simulations have been identified following the procedure described in the Methods section, where also the network parameters corresponding to the proposed results are summarized.

We resorted to this strategy as the dynamics represented by Eq (14) is known to display a remarkable agreement between theory and simulations for spiking neuron networks with instantaneous synaptic transmission and distribution of delays [15, 29, 32].

#### Match between input-output gain functions

**Fig 3:**
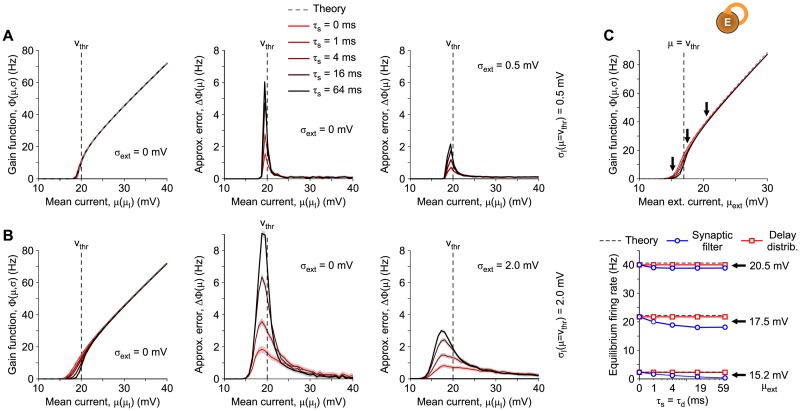
Match between theory and simulations in the input-output gain function of LIF neurons. Gain functions Φ for *N* = 4000 LIF neurons (uncoupled and coupled), obtained by varying the total mean current *μ* = *μ*_*I*_ + *τ*_*m*_*I*_*ext*_/*C*_*m*_ for different *τ*_s_ and different size *σ*_*I*_ of the synaptic current fluctuations: (A) $\sigma_{I}{|}_{\mu =v_{\text{thr}}}=0.5$ mV and (B) $\sigma_{I}{|}_{\mu =v_{\text{thr}}}=2.0$ mV. In the explored range of *μ*, *σ*_*I*_ varies between 74% and 138% of its value at *μ* = *v*_thr_. Left panels in (A-B): theoretical Φ_0_ from 0-th order approximation (*τ*_s_ = 0, gray dashed curves) and measured $\Phi_{\tau_{s}}$ for *τ*_s_ ∈ {0, 1, 4, 16} ms (reddish curves) from single-neuron simulations. Central and right panels in (A-B) show the differences $\Delta \Phi =\Phi_{\tau_{s}}-\Phi_{0}$, either including (right) or excluding (middle) an additional unfiltered white noise with $\sigma_{ext}=\sigma_{I}{|}_{\mu =v_{\text{thr}}}$. Only the three curves with *τ*_s_ > 0 are shown as the simulations with *τ*_s_ = 0 largely overlap the x-axis due to the remarkable agreement with the theoretical Φ_0_. (C) Gain functions $\Phi_{\tau_{s}}$ *versus* the mean external current *μ*_*ext*_ for a network of excitatory neurons. Network topology and neuron parameters as in Panel (B) with *σ*_*ext*_ = 2.0 mV, but with 50% of recurrent synapses (the others receive Poissonian spike trains as in (B)). Thick arrows: values of *μ*_*ext*_ = {15.2, 17.5, 20.5} mV driving the network from noise- to drift-dominated regimes, respectively. Bottom panel, steady-state firing rates (fulfilling the equilibrium condition Φ_0_(ν*) = ν*) of the network with non-instantaneous synaptic transmission (blue plots) and distribution of axonal delays (red plots) at the highlighted *μ*_ext_. Shaded strips in all panels: mean ± SEM from 10 independent simulations (see Methods for further details and parameters).

As a first evaluation of the effectiveness of the low-dimensional description derived above, we inspect the simplest condition given by the asymptotic firing rate of isolated neurons with or without a synaptic filtering of the incoming spike trains. In the absence of recurrent connectivity and under stationary condition, from Eq (12) the firing rate *ν* is given for any *τ*_s_ by the single-neuron input-output gain function Φ_0_(*μ*, *σ*). In Fig 3, we show the results of this test by comparing the steady-state firing rates of LIF neurons measured from single-neuron simulations and their theoretical input-output gain function Φ_0_(*μ*, *σ*) [27, 33, 34]:

$$\begin{array}{c} \frac{1}{\Phi_{0}\left(\mu ,\sigma \right)}=2\;\tau_{m}\int_{\frac{v_{\text{res}}-\mu }{\sigma }}^{\frac{v_{\text{thr}}-\mu }{\sigma }}dy\;e^{y^{2}}\int_{y}^{\infty }dz\;e^{-z^{2}}\;, \end{array}$$ (16)

where *μ* = *μ*_*I*_ + *τ*_*m*_*I*_*ext*_/*C*_*m*_ is the total mean input current and $\sigma^{2}=\sigma_{I}^{2}+\sigma_{ext}^{2}$ is the total variance. Each curve has been obtained by changing the rate *ν* of the external source of Poissonian spikes, such that both *μ*_*I*_ and *σ*_*I*_ change simultaneously, according to Eq (2).

The 0-th approximation provided by Eq (16) (gray dashed curves in Fig 3-left) perfectly overlaps with the simulation of a single LIF neuron incorporating instantaneous synaptic transmission (*τ*_s_ = 0, red curves), confirming the validity of the diffusion approximation. However, the output firing rates at fixed input *μ* change with increasing *τ*_s_, as described in [8, 11]. Such discrepancy can be better appreciated from the central panels of Fig 3, where the differences ΔΦ between Φ_0_ and the gain functions $\Phi_{\tau_{s}}$ from simulations are shown to mainly increase with *τ*_s_. This could seem in contradiction with the fact that our low-dimensional reduction is independent of the synaptic filtering timescales arising in the steady-state firing rate of isolated neurons, but it is not. Indeed, by comparing two examples at relatively small and large size *σ*_*I*_ of the synaptic current fluctuations (Fig 3A and 3B, respectively), the equivalence is recovered as long as *σ*_*I*_ ≪ *v*_thr_, according to the assumption at the basis of our 0-th order approximation.

Note that the largest discrepancies are found in a relatively small range of the mean current *μ* around *v*_thr_, where synaptic fluctuations have a major role in the spike emission process and this range shrinks with decreasing *σ*_*I*_. Outside this range of *μ*, our 0-th order approximation is still valid to describe the steady-state firing rate of single-neurons both under noise- and drift-dominated regime.

Finally, we remark that the discrepancy between theory and simulations can be further reduced by adding the white noise with size *σ*_ext_ > 0. We introduced this additional input in the limit *σ*_*ext*_ → 0 in Eq (4) to safely manage the boundary conditions in the spectral expansion of the one-dimensional operator $L_{xy}$. However, it has been suggested to effectively model several neuronal features like the ionic channel stochasticity [35, 36] and the effect of thermal noise [37]. As a result, under this biologically plausible condition, the match between theoretical input-output gain function and simulations is recovered also for relatively large *σ*_*I*_ (Fig 3B-right).

As a second test, we consider the same network as above, with the same total number of average synaptic inputs per neuron, but with 50% of synaptic contacts from external Poisson-like excitatory neurons and 50% of recurrent synapses. Under stationary condition, we obtain results similar to those observed in the absence of recurrent connectivity: Fig 3C shows the gain functions $\Phi_{\tau_{s}}$ for this network, plotted *versus* the mean external current *μ*_*ext*_ (upper panel) and the steady-state firing rates of the network, satisfying the equilibrium condition Φ(ν*) = ν* [25], plotted *versus* *τ*_s_ = *τ*_d_ (lower panel). The abscissa *μ*_*ext*_ is the sum of two mean currents: the white noise with drift and the exogenous Poissonian input. Notice that the average firing rate *ν*_0_ when delay distributions are incorporated in simulation (red curves in lower panel) does not change by varying *τ*_d_. This is an expected result, as under stationary conditions the input firing rate $\tilde{\nu }$ in Eq (14) coincides with the equilibrium value independently of *τ*_d_. It is also apparent that the mean-field equilibrium point ν* (dashed lines) mildly overestimates the simulated equilibrium point *ν*_0_ when both a delay distribution (red lines) or synaptic filters (blue lines) are incorporated. This is due to the assumptions underlying the diffusion and the mean-field approximations. Increasing the number of synaptic contacts per neuron and the network size *N* by keeping unchanged drift and diffusion coefficients of the related Langevin equation leads to reduce the difference |*ν*_0_ − ν*|. Finally, due to the changes in the input-output gain function Φ_0_ shown in the top panel, the measured mean firing rates *ν*_0_ in the network with non-instantaneous synaptic transmission are different from those with distribution of axonal delays, and the difference increases with *τ*_s_ = *τ*_d_ [8, 10, 11]. This trend is particularly apparent around the critical value *μ* = *v*_thr_ (see the case *μ*_*ext*_ = 17.5 mV in the bottom panel), where our 0-th order perturbative approach is less accurate unless *σ*_*I*_ ≪ *v*_thr_. We remark that the equivalence between delay distribution and synaptic filtering holds in both noise- (subthreshold) and drift-dominated (suprathreshold) regimes, and it is less accurate only at the transition between these two conditions.

#### Responses to a time-varying input

In the comparison between steady-state firing rates, neither delay distribution nor out-of-equilibrium conditions have been explicitly tested as the input-output gain function is a single-neuron feature and the adopted input currents had stationary mean and variance.

**Fig 4:**
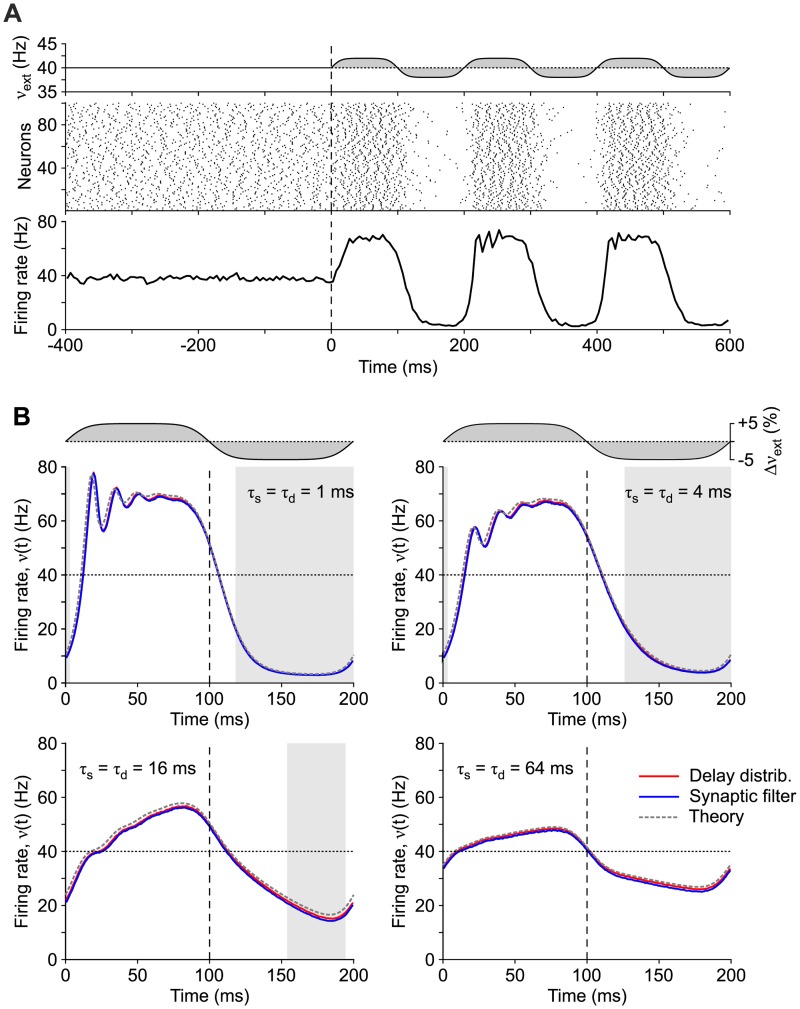
Network response to a periodic step-wise input. (A) Example of response of a network of LIF neurons to periodic modulation of *ν*_*ext*_(*t*) = (1 + Δ*ν*_*ext*_ erf(2 sin(2*πt*/*T*))) 40 Hz with *T* = 200 ms and Δ*ν*_*ext*_ = 0.05 (top). Spiking activity and firing rate *ν*(*t*) (middle and bottom, respectively). Same network as in Fig 7A at drift-dominated regime with instantaneous synaptic transmission and a distribution of axonal delays with *τ*_d_ = 1 ms. (B) Average responses to a stimulation period (200 ms) of (blue curves) the same LIF neuron network with synaptic filters at different timescales *τ*_s_ = 1, 4, 16, 64 ms and (red curves) the equivalent networks with distributions of delays (*τ*_d_ = 1, 4, 16, 64 ms). White and gray shaded intervals mark the time when neurons in the network work in drift- (*μ*_*I*_ + *μ*_*ext*_ > *v*_thr_) and noise-dominated (*μ*_*I*_ + *μ*_*ext*_ < *v*_thr_) regime, respectively. Averages are computed across 300 periods, removing a first transient response (1 s). A grand average of this *ν*(*t*) is eventually computed across 10 networks with same mean-field parameters but randomly different connectivity matrices. Gray dashed curves: theoretical *ν*(*t*) resulting from the numerical integration of the one-dimensional FP Eq (13).

To overcome this limited testing condition, here we further test the first-order statistics of the firing rate, by simulating the network of LIF neurons shown in Fig 7A with a time-varying input (Fig 4A). To this purpose, the rate of external spikes *ν*_*ext*_(*t*) was modulated as a periodic wave with period *T* = 200 ms. Average responses of the firing rate *ν*(*t*) across a stimulation period are shown in Fig 4B for networks with the same mean-field parameters but different time scales *τ*_d_ = *τ*_s_. Remarkably, in all conditions, the average firing rate of each network with synaptic filters (blue curves) widely overlaps with the one computed in the equivalent network incorporating a suited distribution of transmission delays (red curves) and also with the numerical integration (using the Python library described in [38]) of the one-dimensional FP Eq (13) equivalent to Eqs (12) and (14). We remark that very similar results can be obtained by using only the two slowest modes of the FP operator spectrum.

The similarity of the response in the theoretical *ν*(*t*) and the two simulated networks is apparent for any *τ*_s_ = *τ*_d_ ranging from 1ms to 64 ms, during both the fast transient elicited by the sudden changes of *ν*_*ext*_ (at *t* = 0 and 100 ms), and the relaxation phases when the networks drift towards a new equilibrium point. The latter effect is not a surprise, being due to the expected low-pass filtering. However, notice the reduction of the time lag in crossing 40 Hz (firing rate of the unperturbed networks) as *τ*_d_ = *τ*_s_ increases. For networks with synaptic filtering, when the input suddenly changes, the non-instantaneous synaptic transmission leads to have a uniform shift of the probability density *ρ*_*x*_(*x*, *t*) towards the emission threshold (towards right in Fig 1). As a consequence, firing rates can display arbitrarily fast reactions, which are even faster as *τ*_s_ increases [6, 9]. Conversely, networks with transmission delays do not express the same mechanism, as they have vanishing probability densities for *V* → *v*_thr_. Here, the coexistence of slow and fast dynamics is likely due to the fact that, for *τ*_d_ long enough compared to *T*/2, *ρ*_*x*_(*x*, *t*) has not enough time to approach its asymptotic value. As a consequence, just before the input has a step transition, a fraction of neurons (those with the longest pre-synaptic transmission delays) has still memory of the previous stimulation phase. This subset of neurons, whose size increases with *τ*_d_, are the first to be primed, thus leading the whole network to rapidly react to *ν*_*ext*_ changes.

We remark that, as *ν*(*t*) changes across the stimulation period, not only *μ*_*I*_, but also $\sigma_{I}^{2}$ changes accordingly. This notwithstanding, the match of the three curves is remarkably good under both drift- and noise-dominated regimes (white and gray shaded intervals in Fig 4B, respectively), witnessing the quite general validity of the proposed approach.

#### Equivalence in the asynchronous state of finite-size networks

As the equivalence between synaptic filter and delay distribution holds for the mean *ν*(*t*), here we evaluate the equivalence validity also for the second-order statistics of the firing rate around a stable equilibrium point. To this purpose, we inspect the activity in the presence of an endogenous noise in a network of a finite number *N* of neurons trapped into a stable asynchronous state. As previously done in [15], finite-size fluctuations can be incorporated into Eqs (12) and (14) as an additive forcing term to the expansion coefficient dynamics, giving rise to an equation for the firing rate *ν*_*N*_(*t*) of a finite pool of neurons (see Methods for details). The resulting dynamics can be linearized around the equilibrium point ν* = Φ_0_(ν*). This allows to compute the Fourier transform *ν*_*N*_(*ω*), and from it the power spectral density *P*(*ω*) = |*ν*_*N*_(*ω*)|^2^, which turns out to be

$$\begin{array}{c} P\left(\omega \right)=\frac{1+2\;\text{Re}[{\vec{f}}_{0}\cdot {\left(i\omega I-\Lambda_{0}\right)}^{-1}{\vec{\psi }}_{0}]}{{\left|1-[\Phi_{0}^{\prime }+i\omega {\vec{f}}_{0}\cdot {\left(i\omega I-\Lambda_{0}\right)}^{-1}{\vec{w}}_{0}]r\left(i\omega \right)\right|}^{2}}\frac{\nu^{*}}{N}\;. \end{array}$$ (17)

Here, **I** is the identity matrix, all the coefficients depending on *ν*(*t*) are now constants computed at *ν*(*t*) = ν*, $\Phi_{0}^{\prime }=\partial_{\nu }\Phi_{0}$ and *r*(*iω*) is the ratio between the Fourier transforms of *μ*_*y*_ and *μ*_*I*_, which results to be *r*(*iω*) = 1/(1 + *iωτ*_s_). Note that *r*(*iω*) coincides with the Fourier transform *ρ*_*d*_(*iω*) of the delay distribution (15) with *τ*_d_ = *τ*_s_. If we consider an additional fixed transmission delay *δ*, this transform generalizes to *r*(*iω*) = exp(−*iωδ*)/(1 + *iωτ*_s_) [15, 29].

**Fig 5:**
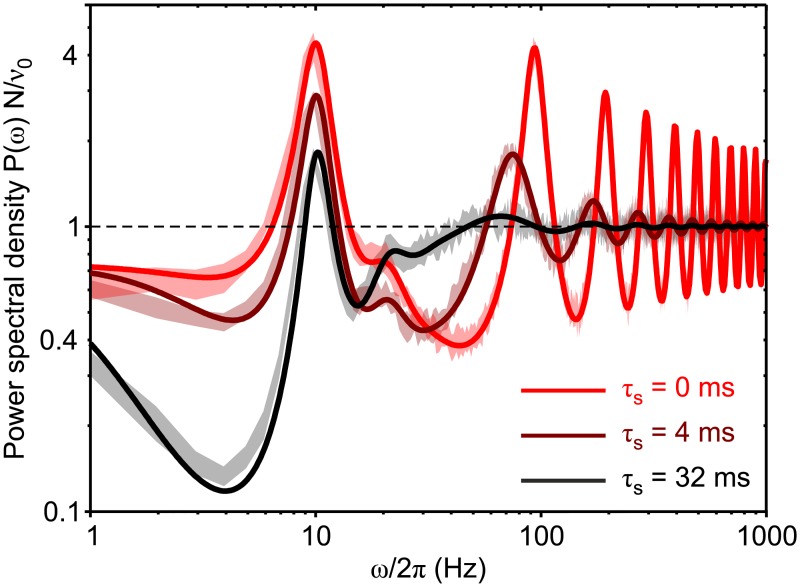
Match between theory and simulations in networks of VIF neurons working in drift-dominated regime with different synaptic decay times *τ*_s_. Power spectral densities *P*(*ω*) of the finite-size network firing rate *ν*_*N*_(*t*) are shown for *τ*_s_ = {0, 4, 32} ms. All networks have a stable equilibrium point at ν* = 10 Hz, and are composed of *N* = 2000 excitatory VIF neurons. Synaptic matrices are random with connection probability *C*/*N* = 5% and average synaptic efficacy *J* = 7.510^−3^ *v*_thr_. Spikes are delivered to the postsynaptic targets with a fixed transmission delay *δ* = 10 ms (see Table 1 for other parameters). Solid lines: theoretical *P*(*ω*) from Eq (17) computed relying on the first 4096 modes of the FP operator spectral expansion. Shaded strips: mean ±3 SEM (standard error mean) of *P*(*ω*) from 10 simulations with different random synaptic matrices and same mean-field parameters. Power spectra are normalized by *N*/*ν*_0_, where *ν*_0_ is the average in time of *ν*_*N*_(*t*) and *ν*_0_ = ν* for theoretical *P*(*ω*).

We compared this theoretical result with the *P*(*ω*) estimated from simulations of networks composed of simple IF neurons with synaptic filtering (see Methods for details), finding a remarkable agreement (Fig 5). Here we resorted to the VIF neuron model [21], an extended version of the widely used PIF neuron [39], for its amenability to analytical treatment [15] (see Methods for details).

As stated above, the theoretical power spectral density has an equilibrium point ν*. On the other side, the microscopic simulations provide a firing rate *ν*_*N*_(*t*) whose mean value (averaged over 10 simulations with different random synaptic matrices and same mean-field parameters) is *ν*_0_. Each power spectral density plot provides information about the frequencies at which the *ν*_*N*_(*t*) variations are stronger.

The theoretical *P*(*ω*) from Eq (17) is computed on the first 4096 modes/eigenfunctions of the FP operator. The large number of modes is justified by the need of capturing the network behavior over a wide frequency range, characterized by the presence of many narrow resonant peaks.

Besides the good matching between theory (solid lines) and simulations (shaded strips), it is interesting to note how resonant peaks are differently affected by increasing *τ*_s_. Not surprisingly, high-*ω* peaks are broadened due to the low-pass filtering of synaptic transmission. These resonances are related to the synaptic reverberation of spiking activity, which gives rise to the so-called *transmission* poles in the linearized dynamics [15, 40]. In an excitatory network working in drift-dominated regime as in Fig 5, these are expected to be found at frequencies multiple of the inverse of the average time needed by a presynaptic spike to affect postsynaptic potential, here roughly 1/(*δ* + *τ*_s_). On the other hand, the low-*ω* peaks do not display any shift in frequency, although their power is broadened as well. This is because such peaks are due to the so-called *diffusion* poles in the linearized dynamics [15, 41]. They occur when neurons emit spikes at drift-dominated (suprathreshold) regime. In this regime, the distribution of the inter-spike intervals is narrow and resonant peaks emerge at *ω*/2*π* multiples of ν* (equal to 10 Hz in Fig 5). We remark that, due to the shifting at low-*ω* of the transmission peaks, a constructive interference with diffusion peaks may occur. This explains the non-monotonic change of power of the second diffusion peak at 20 Hz in Fig 5. Indeed, its power decreases when *τ*_s_ is increased from 0 ms to 4 ms, as expected, whereas due to the mentioned interference it is heightened for *τ*_s_ = 32 ms. This is because the lowest transmission peak in this case is expected to be found at *ω*/2*π* = 23.8 Hz, not too far from 2ν* = 20 Hz.

#### Equivalence under noise- and drift-dominated regime

To further test the equivalence, we investigated whether this match worked well for networks of neurons not only under drift-dominated spiking regime, as shown in Fig 5, but also under noise-driven regimes. Indeed, synaptic filtering may have significantly different effects on the steady-state firing rate in these two regimes [9, 11], as shown in Fig 3.

**Fig 6:**
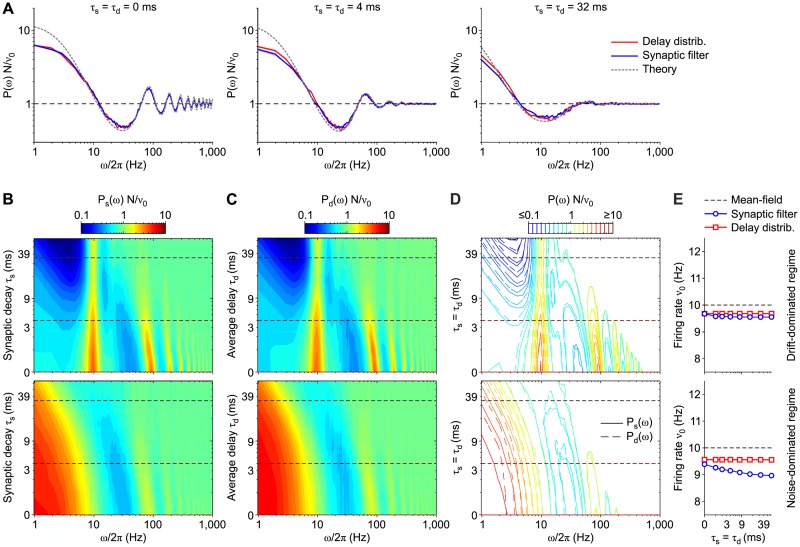
Equivalence of non-instantaneous synaptic transmission and distribution of synaptic delays in a network of VIF neurons. (A) Power spectral densities *P*(*ω*) of *ν*(*t*) obtained for *τ*_s_(= *τ*_d_) = {0, 4, 32} ms in an excitatory VIF neuron networks under noise-dominated regime with either non-instantaneous synaptic transmission (blue) or distribution of transmission delays (red). Gray dashed curves: theoretical *P*(*ω*) from Eq (17) computed relying on the first 256 modes of the FP operator spectral expansion. (B) Power spectral density *P*_*s*_(*ω*) of the simulated network activity in the case of non-instantaneous synaptic transmission, varying the synaptic time constant *τ*_*s*_. Dashed lines: *P*(*ω*) sections plotted in Fig 5 and in panel A. (C) *P*_*d*_(*ω*) for networks with an exponential distribution of spike transmission delays and with instantaneous synaptic transmission, varying the delay time constant *τ*_d_. In B and C, top and bottom panels show the results for the network set in an asynchronous state in which neurons are in a drift- (top) and noise-dominated (bottom) regime, respectively. (D) Iso-power curves from panels B (solid lines) and C (dashed lines). (E) Average firing rate *ν*_0_ from the simulations used for the left panels with non-instantaneous synaptic transmission (blue plots) and distribution of axonal delays (red plots). Error bars representing SEM are not visible. The not specified network parameters are as in Fig 5 (see Table 1). Also here, power spectra are averages across 10 simulations with different random synaptic matrices and same mean-field parameters.

Fig 6A shows for comparison the power spectral densities *P*(*ω*) of the firing rate *ν*(*t*) from an excitatory VIF neuron network working under noise-dominated regime by varying *τ*_s_(= *τ*_d_), in which either non-instantaneous synaptic transmission (blue curves) or a distribution of transmission delays (red curves) was incorporated. The theoretical *P*(*ω*) from Eq (17) (gray dashed curves) overlaps the simulation results for a wide range of *ω*. Notice that we used the first 256 modes of the FP operator spectrum, i.e., 16 times less modes than for the drift-dominated case: this points out the greater effectiveness of our approach in the noise-dominated regime. It is also important to remark that the discrepancy observable at low-*ω* is not due to a failure of our approximation, as it occurs also at *τ*_s_ = *τ*_d_ = 0 ms, when synaptic transmission is instantaneous. This indeed is related to the mean-field approximation, which overestimates the firing rate of the equilibrium point, as explained more in detail later in this subsection.

Fig 6B (non-instantaneous synaptic transmission) and Fig 6C (distribution of transmission delays) show the same comparisons for a larger set of *τ*_s_ = *τ*_d_ values (in logarithmic scale), corresponding to networks working in noise- (bottom panels) or drift-dominated (top panels) regimes; in this case, the power spectrum amplitude is color-coded. The power spectral densities in panel A correspond to the cuts marked by dashed horizontal lines in (bottom) panels B and C. A remarkable agreement between simulations in the two tested conditions is confirmed by Fig 6D, showing iso-power curves from panels B (solid lines) and C (dashed lines): the equivalence of non-instantaneous synaptic transmission and distribution of axonal delays is witnessed by the tight overlapping of solid and dashed iso-power curves for a wide range of filter timescales.

The same good matching between these two network types is apparent also under noise-dominated regime (bottom panels). As expected in this regime, the diffusion poles become real numbers [15] and resonant peaks at frequencies multiple (by *ω*/2*π*) of the equilibrium firing rate ν* disappear, although the high-*ω* resonances corresponding to transmission poles remain almost unaffected by this change of regime. We point out that widening the filtering time window (i.e., increasing *τ*_s_ and *τ*_d_) leads to almost flat power spectra (see Fig 5), compatibly with the flattening of the response amplitude of isolated neurons receiving colored noise as input currents [9, 10].

As shown in Fig 1B–1D, the section of *ρ*(*x*, *y*, *t*) close to firing threshold *v*_thr_ shrinks when *τ*_s_ increases from 4 ms to 64 ms. In our perturbative approach this dependence is completely neglected, as the distribution is assumed to be a Dirac’s *δ*.

We remark that the shown comparisons rely on normalized spectra, i.e. *P*(*ω*)*N*/*ν*_0_. On the one hand, this means that power spectra shapes do not depend on the particular transmission protocol implemented in the network. On the other hand, this does not guarantee, as shown for the LIF neuron case, that average firing rates *ν*_0_ are the same and do not change with the protocol-related timescales. To address this issue, in Fig 6E the measured *ν*_0_ are compared, showing features completely similar to those observed in Fig 3 for the LIF neuron model.

Notice that the discrepancy between theory and simulations (*ν*_0_ and ν*, respectively) explains the differences found at low-*ω* in Fig 6A. Indeed, such difference leads to have different slopes of the gain function ($\Phi_{0}^{\prime }\left(\nu_{0}\right)\neq \Phi_{0}^{\prime }\left(\nu^{*}\right)$), which in turn affect differently the power spectrum at low-*ω* as $P\left(\omega =0\right)\propto {\left(1-\Phi_{0}^{\prime }\right)}^{-2}$.

Since we already checked the accuracy of the theoretical *P*(*ω*), in the rest of the paper we will focus on the power spectral densities estimated from microscopic simulations. Taking as reference these simulations instead of Eq (14) in the comparison with the supposedly equivalent networks with synaptic filtering has indeed the advantage of allowing to test our theoretical prediction also in neuron models for which analytical expressions for the eigenvalues and eigenfunctions of the FP operator are not available, as in the case of exponential IF (EIF, [42]) neurons. Moreover, this strategy allowed us to overcome the possible issue related to computing a large number of modes of the FP operator spectrum for more realistic single-cell models.

#### Independence from spiking neuron models

The developed theory is of general applicability, i.e. it applies to a wide range of spiking neuron models. As a result, the equivalence proved for a network of simplified VIF neurons together with the theoretical expression for the power spectra of *ν*(*t*) are both expected to hold also for networks of more realistic single-cell models like the LIF and the EIF neurons.

**Fig 7:**
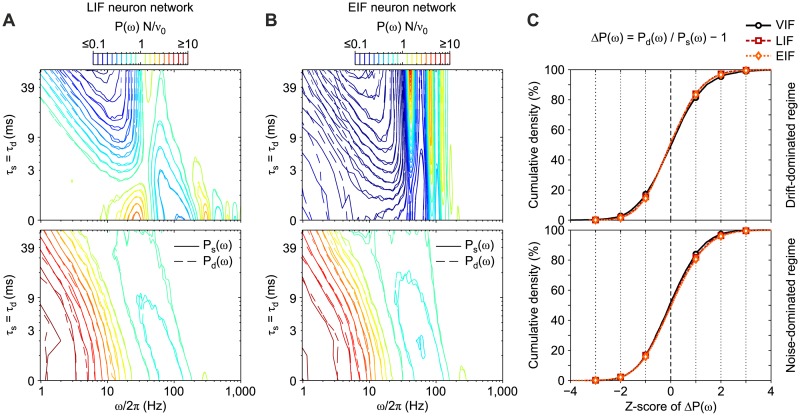
Equivalence between synaptic filtering and delay distribution in networks with different types of IF neurons. Match between relative power spectral densities *P*(*ω*)*N*/*ν*_0_ in networks of leaky (A) and exponential (B) integrate-and-fire excitatory neurons (LIF and EIF, respectively) with non-instantaneous synaptic transmission (solid lines) and distributions of synaptic delays (dashed lines). Network parameters are set in order to have an asynchronous state with average firing rate *ν*_0_ = 40 Hz. Contour lines as in Fig 6D. *P*(*ω*) are averages across 10 simulations with different random synaptic matrices and same mean-field parameters. (C) Cumulative distribution of relative differences Δ*P*(*ω*) = *P*_d_(*ω*)/*P*_s_(*ω*) − 1 across different time scales (*τ*_s_ and *τ*_d_) and Fourier frequencies *ω* from panels A and B for LIF (red) and EIF (orange) neuron networks, respectively. As a reference, also the same cumulative distribution for VIF neuron networks (obtained from *P*(*ω*) in Fig 6D) is plotted (black). Δ*P*(*ω*) are measured as *z*-scores, i.e. averages divided by standard deviations of values across simulations. Top and bottom panels: networks working under drift-dominated and noise-dominated regime, respectively (see parameters in Tables 2 and 3).

To verify this expectation, we directly simulated networks of LIF and EIF neurons with a mean-field stable equilibrium point at ν* = 40 Hz. In Fig 7 the mean *P*(*ω*)*N*/*ν*_0_ for these kinds of networks is compared both under drift-dominated (top panels) and noise-dominated (bottom) regimes. A remarkable agreement is apparent for both LIF and EIF neuron networks (Fig 7A and 7B, respectively), confirming the generality of the developed approach. To further test the absence of any bias due to the specific neuron model chosen, we computed the relative differences between the power spectral densities when non-instantaneous synaptic filtering (*P*_s_) and transmission delay distribution (*P*_d_) are taken into account. The cumulative distributions of these discrepancies across all tested time scales (*τ*_s_ = *τ*_d_) and Fourier frequencies (*ω*/2*π*) are shown in Fig 7C. Interestingly, no significant differences are visible for the three chosen models (VIF, LIF, EIF), further proving that in all regimes (drift- and noise-dominated) our dimensional reduction does not depend on the specific single neuron dynamics.

Starting from this, it is not surprising to note here that spectral features similar to those highlighted in Fig 6 for VIF neuron networks are also displayed by LIF and EIF neuron networks. More specifically, resonant peaks at multiple frequencies of *ν*_0_ under drift-dominated regimes and resonances at higher-*ω* due to the transmission poles—i.e., those related to the average spike transmission delay—are also visible in both Fig 7A and 7B in LIF and EIF neuron networks, respectively.

#### Equivalence beyond the asynchronous state

**Fig 8:**
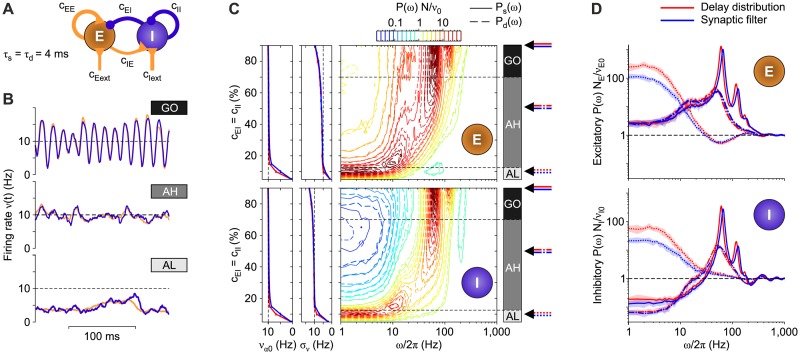
Comparison between multi-modular networks of inhibitory and excitatory LIF neurons with either synaptic filtering or transmission delay distribution. (A) Sketch of the multi-modular network. (B) Three examples of firing rate time evolution for each network stable state. Plum and orange curves represent inhibitory and excitatory neuronal populations, respectively, with synaptic filter. (C) Left plots: mean (*ν*_*α*0_, *α* = E, I) and standard deviation (*σ*_*ν*_) of the instantaneous firing rate across time (binning size of 0.1 ms, discarding the first second of the time series) for inhibitory (bottom panels) and excitatory (top panels) populations. Right plots: match between relative power spectral densities *P*(*ω*)*N*/*ν*_*α*0_ for excitatory (top panel) and inhibitory (bottom panel) populations with non-instantaneous synaptic transmission (solid lines) and distributions of transmission delays (dashed lines). Contour lines as in Fig 6D. Different states are obtained by increasing the connection probability with pre-synaptic inhibitory neurons, namely *c*_*αI*_ ≡ *c*_EI_ = *c*_II_ (see text for details). The spectra *P*(*ω*) are averages across 10 simulations with different random synaptic matrix realizations. Dashed horizontal lines roughly mark the bifurcations. Rightmost arrows point out the values of *c*_*αI*_ corresponding to the examples displayed in panels B and D. (D) Comparisons between relative spectra from networks with delay distribution (red lines) and synaptic filtering (blue lines) for the three network states AL (dotted lines), AH (dashed-dotted lines), and GO (solid lines), for excitatory (top panel) and inhibitory (bottom panel) populations.

So far, we tested the validity of the equivalence between non-instantaneous synaptic transmission and delay inhomogeneity in linearizable dynamical regimes like the asynchronous state and in purely excitatory networks. To further assess the generality of our theoretical results, we simulated a multi-modular network containing an inhibitory (I) and an excitatory (E) population of LIF neurons (Fig 8A, see Methods), with the purpose of analyzing its dynamical behaviors by changing a bifurcation parameter.

In this specific example network, the bifurcation parameter is the percentage *c*_*EI*_ = *c*_*II*_ ≡ *c*_*αI*_ of outward inhibitory synaptic connections per neuron. To compensate for these changes in the inhibitory component, the synaptic efficacies of both recurrent and external excitatory neurons are progressively increased: this allows to keep an equilibrium point ν* = Φ_0_(ν*) at the same relatively high firing rate $\nu_{E}^{*}=\nu_{I}^{*}=10$ Hz for both populations, regardless of its stability (see details in Methods). For low enough *c*_*αI*_, this equilibrium point is unstable and only an asynchronous irregular state at low firing rate (denoted as AL) can be reached by both populations, as shown in Fig 8B-bottom for the E-I network with *c*_*αI*_ = 10% and non-instantaneous synaptic transmission. A suited increase of *c*_*αI*_ stabilizes the high-frequency asynchronous state (denoted as AH), in which the neurons fire at the equilibrium rate of 10 Hz (Fig 8B-middle, *c*_*αI*_ = 50%). A stronger synaptic inhibition eventually leads the E-I network to display synchronous firing with coherent global oscillations (GO) of the spiking activity (Fig 8B-top, *c*_*αI*_ = 90%).

We compared networks with both synaptic filtering and delay distribution across this repertoire of dynamical regimes, by making extensive simulations on a finer grid of *c*_*αI*_ values, by using the same mean-field parameters (Fig 8C). In all cases the networks undergo the same state transitions at the same critical *c*_*αI*_ values. More specifically, the value *c*_*αI*_ ≈ 12.5% marks a transcritical bifurcation where the low-frequency and the 10-Hz equilibrium points exchange their stability and the transition from AL to AH occurs. In both networks, the mean firing rate *ν*_0_ of the AL state increases with its standard deviation *σ*_*ν*_ (Fig 8C-left) until the 10-Hz equilibrium point corresponding to the AH state becomes the lowest stable equilibrium point. This point undergoes a supercritical Hopf bifurcation at *c*_*αI*_ ≈ 70%, where the network has a transition from the AH to the GO state. The resulting synchronous oscillations of the firing rate have an amplitude that increases with *c*_*αI*_. This is why *σ*_*ν*_ increases and *ν*_0_ remains unchanged at 10 Hz. Even in this case, the first two moments of the firing rate in networks with both non-instantaneous synaptic transmission (blue) and distribution of axonal delays (red) match almost perfectly.

This close resemblance is also apparent in the relative power spectra *P*(*ω*)*N*/*ν*_0_ shown in Fig 8C-right, although significant changes in the power distribution emerge when the network state has a transition. To better appreciate the comparison between non-instantaneous synaptic transmission (solid lines) and distributions of axonal delays (dashed lines), Fig 8D (where *c*_*αI*_ = {10, 50, 90}%, as in Fig 8B) shows three paradigmatic relative spectral densities. As expected, *P*(*ω*) for the network in the AL state close to the bifurcation (red and blue dotted curves) displays a large peak at low-*ω* due to the slow fluctuations of the firing rate determined by the weakening of the 10-Hz attractor stability. As $P\left(\omega =0\right)≃{\left(1-\Phi_{0}^{\prime }\right)}^{-1}$ (see Eq (17)), the small but significant differences between the spectra at low-*ω* for non-instantaneous synaptic transmission (blue) and distributions of axonal delays (red) have to be attributed to the different shape of the input-output gain functions Φ_0_. Indeed, our approximated Φ_0_ overestimates the steady-state firing rate when *τ*_s_ > 0 (see Fig 3) in a relatively narrow range of the mean input current *μ* around *v*_thr_, thus affecting also the slope $\Phi_{0}^{\prime }$. This difference is also responsible for the small discrepancies between the measured *ν*_0_ and ν* as in Fig 6E. Further increasing the recurrent inhibition *c*_*αI*_, in the AH state (dashed-dotted lines) resonance peaks due to transmission poles at *ω*/2*π* ≈ 50 Hz pop up. In the GO state (solid lines) also higher-order harmonics start to be visible in both *P*_*d*_(*ω*) and *P*_*s*_(*ω*). Although the overlap between these spectra is remarkable also in the GO regime, a small unexpected shift of the resonant frequencies is visible, highlighting a mild increase of the oscillation frequency when the synaptic filter is incorporated.

**Fig 9:**
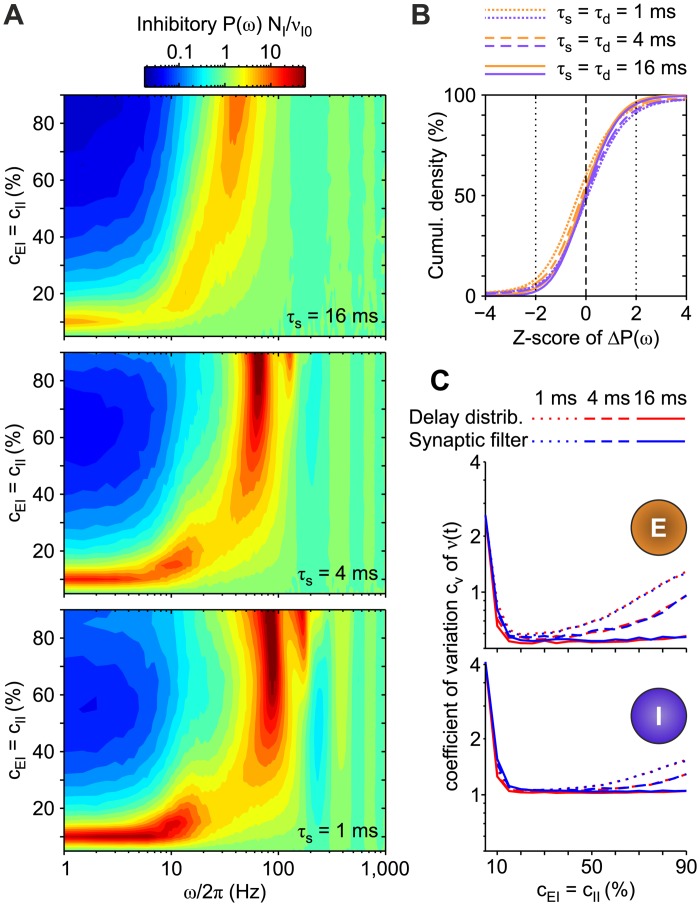
Match between the same multi-modular networks as in Fig 8 for different timescales. (A) Relative power spectra *P*(*ω*) of the inhibitory population activity in the case of non-instantaneous synaptic transmission for three different synaptic time constants (*τ*_s_ = 1, 4, 16 ms). (B) Cumulative distribution (in terms of *Z*-scores) of relative differences between power spectra Δ*P*(*ω*) = *P*_d_(*ω*)/*P*_s_(*ω*) − 1 across Fourier frequencies *ω* for three timescales (*τ*_s_ = *τ*_d_ = 1, 4, 16 ms), as in Fig 7C. Orange and plum curves are associated to excitatory and inhibitory populations respectively, whereas different line styles mark different values of *τ*_s_ = *τ*_d_ (see legend). (C) Coefficient of variation *c*_*v*_ = std(*ν*)/mean(*ν*) of *ν*(*t*) *versus* *c*_*EI*_ = *c*_*II*_ for excitatory (top) and inhibitory (bottom) populations, with non-instantaneous synaptic filtering (blue lines) and distributions of transmission delays (red lines). Different line styles mark different values of *τ*_s_ = *τ*_d_, as in (B).

Similar results were obtained for the same network topology by changing the filtering timescales, as shown in Fig 9. Fig 9A shows relative power spectral densities for the inhibitory pool with non-instantaneous synaptic transmission. As expected, increasing *τ*_s_ from 1 ms to 16 ms (from bottom to top panels, respectively) makes the network more stable, thus shifting at higher *c*_*αI*_ the critical value at which the transition to the GO state occurs and the peaks associated to higher-order harmonics pop up. Larger *τ*_s_ also imply a reduction of the pace of the damped and synchronous oscillations from *ω*/2*π* ≈ 90 Hz to ≈ 40 Hz.

The cumulative distributions of the *Z*-score Δ*P*(*ω*) = *P*_d_(*ω*)/*P*_s_(*ω*) − 1 in Fig 9B show, for the three considered timescales (*τ*_s_ = *τ*_d_), that the differences of spectra display always the same statistics for both excitatory (orange) and inhibitory (blue) populations, similarly to Fig 7C. This equivalence under these conditions is also apparent by comparing the coefficient of variation *c*_*v*_ = std(*ν*)/mean(*ν*) = *σ*_*ν*_/*ν*_0_ for the excitatory and inhibitory pools (top and bottom panels in Fig 9C, respectively), with non-instantaneous synaptic transmission (blue) and exponential delay distributions (red). These plots confirm and complete the results already described in Fig 8C for *τ*_s_ = *τ*_d_ = 4 ms: for low *c*_*αI*_ (network in the AL state) the relative large variability is mainly due to a reduction of *ν*_0_; moreover, *c*_*v*_ remains at its lowest value until the transition from AH to GO occurs. After this bifurcation, it increases with the amplitude of the global oscillation. As remarked above, the Hopf bifurcation value increases with *τ*_s_ = *τ*_d_. In all cases, these plots confirm the sequence of state transitions already pointed out.

Altogether, these results further prove the generality of the theoretical equivalence proposed in this paper, clearly pointing out that both communication protocols give rise to the same collective dynamics not only in a tameable condition like the linearizable asynchronous state, but also for multi-modular networks working in nonstationary states like the GO regime.

#### Limitations of the theoretical approximation

To reduce the dimensionality of the population density dynamics, we introduced a forcing white noise current with an arbitrarily small size *σ*_*ext*_, as in [43]. This allowed us to safely manage the boundary conditions in the spectral expansion. The intrinsic stochasticity of ionic channels of neuronal membrane potential can be the source of this additional white noise current [35, 36]. However, it is important to remark that, in the absence of this external noise, the spectral expansion we used is no longer valid, as it relies on the eigenfunctions of a FP operator with *ρ*(*v*_thr_, *y*, *t*) = 0 [8, 10, 28, 44]. More specifically, if *σ*_ext_ = 0 a different basis for the probability density decomposition must be adopted.

**Fig 10:**
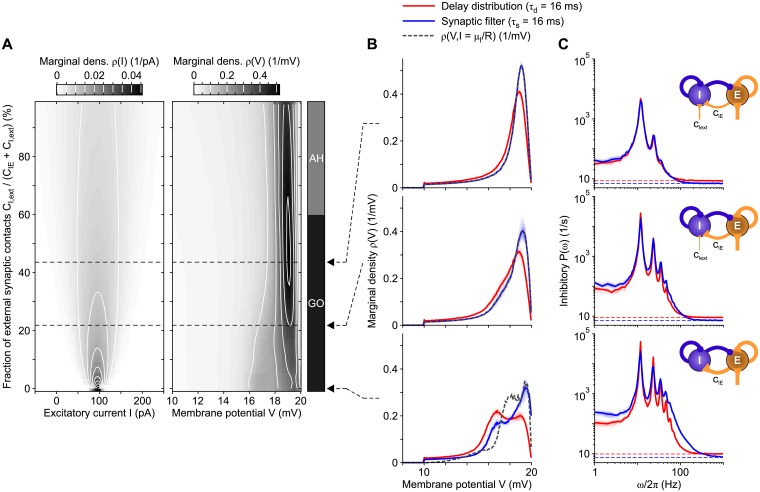
Test of the limitations of the theoretical approximation. (A) Marginal densities of the synaptic current [left, lim_*t*→∞_ *ρ*_*y*_(*I*, *t*) = *ρ*(*I*) = ∫*ρ*(*V*, *I*)*dV*] and the membrane potential [right, lim_*t*→∞_ *ρ*_*x*_(*V*, *t*) = *ρ*(*V*) = ∫*ρ*(*V*, *I*)*dI*] represented also versus the balance *C*_*I*,ext_/(*C*_*IE*_ + *C*_*I*,ext_) between recurrent (synaptically filtered) and external (memoryless) excitatory connections. White lines, iso-density curves. Marginal densities are averaged across 20 randomly chosen inhibitory neurons and 10 simulations with same mean-field parameters but different synaptic matrix realizations. Multi-modular network configuration as in Fig 8, with *c*_*EI*_ = *c*_*II*_ = 1% and *τ*_s_ = *τ*_d_ = 16 ms. (B) Three examples of marginal densities *ρ*(*V*) with different balance between recurrent (filtered) and external (memoryless) excitatory input (see dashed arrows and lines in panel A for the balance value). Blue and red curves: marginal densities from networks with synaptic filtering (as in panel A) and delay distribution, respectively. Gray dashed curves, sections of the density *ρ*(*V*, *I*) centered around the mean synaptic current *I* = *μ*_*I*_/*R* estimated from inhibitory neurons with synaptic filtering. (C) Average power spectral density *P*(*ω*) of the firing rate *ν*_*I*_(*t*) of the inhibitory neurons for the same example networks shown in panel B. The inset network sketches the change in the balance of the recurrent external currents. Color and styles as in panel B. Horizontal dashed lines: asymptotic values (*ν*_*I*0_/*N*_*I*_) of the spectra.

To investigate this aspect more in depth we exploited the multi-modular architecture of the last analyzed network. Indeed, the inhibitory population receives two excitatory contributions: one memoryless from *C*_*I*,ext_ synaptic contacts with Poisson-like external neurons, and another recurrent from the neurons composing the excitatory population. Both kind of synapses share the same synaptic efficacy and all the pre-synaptic (external and recurrent) neurons emit spikes at the same rates. Under mean-field approximation, this implies that, even replacing external with internal synapses, the statistics of the excitatory input received by the inhibitory neurons does not change. As a result, the equilibrium point at 10 Hz persists across variations of the balance between internal and external excitatory input. This is not the case for the equilibrium stability, which is lost as the recurrent contribution increases, giving rise to global oscillations, as shown in Fig 10A. By gradually decreasing the fraction of the external synaptic contacts *C*_*I*,ext_/(*C*_*IE*_ + *C*_*I*,ext_), the peak in the marginal density *ρ*(*I*) shrinks, due to the increasing weight of the low-pass filtered component of the synaptic input *I*(*t*). This shrinking effect is highlighted by the white iso-density curves in Fig 10A-left. Under this condition, a more drift-driven membrane potential *V* is expected and, due to the global oscillations of the firing rate *ν*(*t*), the peak in the marginal density *ρ*(*V*) widens (Fig 10A-right). This spreading of *V* fluctuations can be better appreciated looking at the three selected cuts of *ρ*(*V*) shown in Fig 10B (blue curves). As expected, *ρ*(*V*) in networks with synaptic filters is significantly different from the marginal densities of *V* in the equivalent networks with a distribution of axonal delays (Fig 10B, red curves). This difference is even more apparent when *V* approaches *v*_thr_, as the blue curve does not converge to 0 due to the progressive lack of a diffusive component in the synaptic current. Intriguingly, almost irrespective of these differences, the power spectral density *P*_d_(*ω*) matches remarkably well with *P*_s_(*ω*) (Fig 10C, red and blue, respectively). Even when the memoryless contribution to the synaptic current received by the inhibitory neurons is completely replaced by the recurrent one (Fig 10C-bottom), resonance peaks and the band-pass filtering features widely overlap.

The reasons for the effectiveness of our approximated description are mainly two. Firstly, in networks with synaptic filtering, the marginal density *ρ*(*V*) and the related slice of the full probability density *ρ*(*V*, *I*) centered around the average synaptic current *μ*_*I*_ (dashed gray curves in Fig 10B) are almost indistinguishable, at least for fractions of external synaptic contacts above 20%. In other words, the “slice” at *I* = *μ*_*I*_/*R* (*ρ*(*V*, *μ*_*I*_/*R*)) is well representative of the two-dimensional *ρ*(*V*, *I*). We remark that Eq (12) results from a 0-th order approximation of the current fluctuations which takes into account only this slice. This explains why the equivalence between synaptic filters and delay distributions still holds even when *σ*_ext_ provides a minor contribution to the fluctuations of *I*(*t*).

Secondly, we remind that in our approach the effective population dynamics of the slice at *I* = *μ*_*I*_/*R* is governed by the operator $L_{xy}$, which incorporates an additional diffusion term proportional to *σ*^2^(*y*). This means that the probability density of membrane potentials changes in time according to this one-dimensional FP operator. As a result, Eq (12) actually describes a system with approximately the same firing rate dynamics but with a different *ρ*(*V*, *t*). In other words, we derived an equivalent stochastic diffusion process *V*(*t*) capable to have approximately the same flux of realizations crossing *v*_thr_ (in part due to *σ*^2^(*y*)), but at the same time differing from the original system in the density of *V*. This occurs as *σ*^2^(*y*) and the slope $\partial_{x}{\rho \left(x,t\right)|}_{x=v_{\text{thr}}}$ are adjusted in such a way that the flux of realizations crossing the absorbing barrier at *x* = *v*_thr_ (given by their product, see Eq (13)) is the same as for the 2D case.

The power spectra in Fig 10C allow us to test the limitations of our approximation not only for second-order statistics of the firing activity *ν*(*t*), but also for the first-order statistics, i.e., the mean. Indeed, from Eq (17), the asymptotic power lim_*ω*→∞_ *P*(*ω*) = *ν*_*I*0_/*N*_*I*_ of the inhibitory population provides this information (blue dashed lines in Fig 10C). From this measure, it is rather apparent that no significant changes occur as the diffusion white noise term *σ*^2^(*y*) is progressively replaced by the equivalent filtered synaptic input provided by the excitatory population (*σ*^2^(*y*) ∝ *C*_*I*,*ext*_ → 0). At a first sight, this could seem to contradict what shown in Fig 3C, where the mean-field equilibrium point ν* predicted by our 0-th order approximation is the same for any mean delay *τ*_d_ and differs from the ones measured by incorporating different synaptic filters. Actually, the network displays global oscillations (GO regime) such that the time spent with *μ* ≃ *v*_thr_ is small and, outside a small interval around this critical value, Φ_0_ is a good approximation of $\Phi_{\tau_{s}}$ both under noise- and drift-dominated regimes. Therefore, this result is perfectly in line with the ones shown in Fig 3C.

## Discussion

The main result of this paper is a novel perturbative approach to the population density dynamics of networks with spiking neurons having non-Markovian membrane potentials. This general method consists of a *dimensional reduction* of the two-dimensional population density dynamics arising from the Markovian embedding [2, 3] of the membrane potential evolution when non-instantaneous synaptic transmission is incorporated. The effective one-dimensional description we obtained relies on both an extended mean-field approximation [25] and the assumption of a relatively low synaptic noise (low *σ*_*I*_(*t*)). No additional constraints on the correlation time associated to the incorporated synaptic filtering are imposed. We obtained the same firing rate equation as the one arising from the spectral expansion of the one-dimensional FP equation for the population density in the absence of synaptic filtering [15], but with an important difference: drift and diffusion coefficients, related to the infinitesimal mean and variance of the input currents, respectively, in this case depend on a low-pass filtered version of the instantaneous firing rate of the network.

This theoretical framework led us to obtain our second main result, i.e., the proof that non-instantaneous synaptic transmission and distribution of axonal delays in networks of spiking neurons are equivalent. The conditions of validity of such equivalence are two: the synaptic noise is relatively small (i.e., *σ*_*I*_ ≪ *v*_thr_) and the non-instantaneous post-synaptic currents from incoming spikes have the same time course as the distribution of spike transmission delays across axons and dendrites.

We tested both the generality of the developed theory and the above equivalence through extensive numerical simulations, finding a remarkable agreement with theoretical expectations in a variety of dynamical scenarios, including noise- and drift-dominated regimes, equilibrium states and collective oscillations, excitatory and inhibitory synaptic connections, and for a wide set of spiking neuron models and synaptic timescales. This because in our ‘dynamic’ mean-field approach no constraints on the collective dynamics are imposed, such as those focusing on perturbations of the firing rate around equilibrium points. The nonlinear and state-dependent coupling between the network activity and the membrane potential distribution are fully incorporated into the FP operator through its dependence on the instantaneous firing rate *ν*(*t*). As a result, the time-varying basis composed of the eigenfunctions of this operator effectively follows the evolution of the population density *ρ* at any time resolution keeping small the projections of *ρ* onto the basis axes. Although this basis is shaped by both the specific neuron model (i.e., a specific FP operator) and the neuron coupling structure of the network, the firing rate dynamics derived in Eq (12) does not change and makes this representation of general applicability.

The local-distributed transmission protocol equivalence ultimately allows to map a many-body system with non-Markovian coupled elements onto another many-body system where many Markovian units have delayed interactions [45]. Therefore, the theoretical framework developed in this paper is expected to be exportable to other filtering systems. For instance, the non-Markovian dynamics underlying neuronal spike-frequency adaptation—determined by the activity-dependent modulation of the hyperpolarizing potassium conductances [46–48]—can undergo a dimensional reduction similar to the one introduced here.

**Fig 11:**
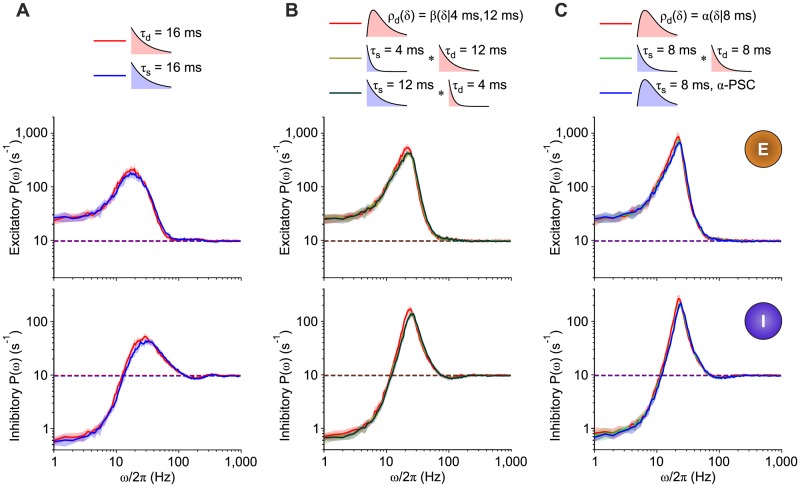
Cascade of synaptic filters and delay distributions. (A) Power spectral densities *P*(*ω*) of the excitatory and inhibitory populations (top and bottom, respectively) firing rate *ν*_*α*_(*t*) (*α* ∈ {*E*, *I*}) of the LIF neuron network shown in Fig 8, with *c*_*αE*_ = 5%, *c*_*αI*_ = 50%, *τ*_d_ = 16 ms (red) and *τ*_s_ = 16 ms (blue). This setting corresponds to a network stable asynchronous state with *ν*_*α*0_ = 10 Hz. The legend (top inset) shows the delay distribution *ρ*_*d*_(*δ*) (red shaded) and the post-synaptic current (PSC, blue shaded), both exponentials in this case. (B-C) Same networks as in (A), in which both the synaptic filter and the delay distribution are simultaneously incorporated keeping fixed the sum *τ*_s_ + *τ*_d_ = 16 ms. (B) Power spectral densities *P*(*ω*) when *τ*_s_ = 4 ms and *τ*_d_ = 12 ms (light green), and *τ*_s_ = 12 ms and *τ*_d_ = 4 ms (dark green). As a reference, a network with instantaneous synaptic transmission and delay distribution *ρ*_*d*_(*δ*) = *β*(*δ*|*τ*_*d*1_, *τ*_*d*2_) resulting from the convolution of two exponentials is also shown (red, see S1 Appendix for details). (C) Power spectral density *P*(*ω*) when *τ*_s_ = *τ*_d_ = 8 ms (green). The other two spectra are from a network with delay distribution *ρ*_d_(*δ*) = *α*(*δ*|*τ*_d_ only (red) and with non-instantaneous synaptic transmission only (blue) giving rise to a PSC elicited by an isolated incoming spike with the same *α*-shape of *ρ*_*d*_(*δ*) (see S1 Appendix for details).

Finally, an important question is how the results proposed in this paper can be used in a broader context. In general, axonal delays and non-instantaneous synaptic transmission coexist in biological neuron networks. So, what happens when axons with given distributions of transmission delays couple on synapses with given time constant? The theoretical approach we developed can be straightforwardly extended to describe also this modeling framework (see S1 Appendix section “Cascade of synaptic filters and delay distributions”), leading to a generalization of Eq (12). This network setting appears to be equivalent to a cascade of two first-order low-pass filters, as shown in Fig 11 (top insets of panels B and C). In turn, these networks have firing rate dynamics equivalent to those obtained with instantaneous synaptic transmission and a suited unimodal distribution of transmission delays. Power spectra of *ν*(*t*) estimated from simulations of E-I networks of LIF neurons (see Fig 11B and 11C) confirm this equivalence.

### Comparison with previous studies

Reducing into an effective one-dimensional Markovian problem the dynamics of a non-Markovian system—which in turn can be embedded into a larger set of coupled Markovian processes—has a long history in statistical physics [2, 7]. The resulting approximation schemes have unavoidable limitations in representing the dynamics of the original non-Markovian problem. Nevertheless, many successes were accumulated in the late 80s [3, 49–53]. Many of these approaches focused on the nonstationary dynamics underlying the specific problem of the first-passage time, thus taking into account an absorbing barrier, similarly to the spike emission threshold in neuronal modeling [3, 53] and in the limit of small noise and/or small correlation times *τ*_s_ [50, 51]. Other perturbative approaches have been worked out to cover the long-*τ*_s_ range [52, 54], but the effectiveness of these approximations is limited by the lack of a proper management of the boundary conditions [7, 28, 44]. Intriguingly, by including the proper absorbing barrier prescription due to the presence of a forcing white noise with arbitrarily small size *σ*_*ext*_ (similarly to [43]), the theoretical derivation we obtained in Eq (12) eventually recovers a representation with time-varying state-dependent diffusion coefficients (see for instance Section V.B of [7]). Indeed, all the coefficients of this Eq (12) depend on a filtered version *μ*_*y*_(*t*) of the total firing rate *ν*(*t*), which from Eq (8) is in turn a nonlinear functional of the probability density *ρ*(*x*, *y*, *t*).

In addition, other dimensional reductions of the FP dynamics arising when synaptic filtering is incorporated in network of spiking neurons have been proposed in the past, to obtain effective kinetic representations amenable to numerical integration [55–57]. They mainly rely on the centered moment closure method usually applied to kinetic problems in statistical physics [16]. Drawbacks of this approach are related to their limited applicability to noise-dominated regimes [57, 58], which are of particular relevance in neuroscience, being associated to activity states with balanced excitation and inhibition [59–61]. Even when some of these limitations are removed by introducing modifications to the standard mean-field approximation [58], this kind of dimensional reduction basically remains a computational method in which, in addition to the dynamics of the centered moments, a one-dimensional partial differential equation has to be numerically integrated. On the contrary, in our theoretical framework, insights on the dynamical properties of the networks can be obtained without strongly relying on numerical integration, similarly to what previously done in [15, 48, 62] by focusing on the slowest modes of the expansion.

Finally, it is important to remark that power spectra and Fourier transfer functions of the population activity in network of spiking neurons and synaptic filtering are typically characterized outside biologically relevant regimes. This is because theoretical approaches are perturbative and target extreme conditions, in which either fast [6, 9, 10] or slow [11, 12] synapses are considered, or in which neurons work under strong drift-dominated (low-noise) regimes [13, 14]. Here, we bridge this gap by presenting a theoretical description valid for both regimes, and for the whole synaptic time scales and significant frequencies (Figs 5–7).

### Other limitations

The developed theoretical description of the network dynamics with synaptic currents is approximated, and as such it is subject to several limitations. Our starting point is a population density approach which, like others [15, 26, 27], relies on both a diffusion and a mean-field approximation. The underlying hypotheses require that each neuron receives a large amount of spikes per unit time, and that postsynaptic currents due to single spikes induce small changes of the membrane potential. These conditions are well satisfied in neocortex [18, 25].

Another strong simplification we implemented was to consider small enough the fluctuation size *σ*_*I*_ of the synaptic currents. Such a low-noise regime gave us the possibility to assume the marginal distribution of the current *I*(*t*) narrowly distributed around its time-varying mean *μ*_*y*_(*t*)/*R*. As a result, the *V* − *I* dynamics were reduced to a one-dimensional FP equation centered around *I*(*t*) = *μ*_*y*_(*t*)/*R*. Rather surprisingly, such a rough approximation, for which the shape of the marginal distribution *ρ*(*I*) is completely neglected, seems to work remarkably well, and this is due to the almost perfect overlap between the marginal distribution *ρ*(*V*) and the one-dimensional density *ρ*(*V*, *μ*_*I*_/*R*), as shown in Fig 10B. The only main discrepancy we found is in the mean firing rate, which our method systematically overestimates (see Figs 3C and 6E). This is an expected error, since synaptic filtering is known to mildly reduce the firing rate of spikes in both the small- [8, 10, 11] and the large-*τ*_s_ [11] limit. This discrepancy should disappear if higher-orders of the centered moment closure were incorporated in our method. Indeed, this would allow to take into account also other features of *ρ*(*I*), such as its standard deviation and skewness.

## Methods

### Power spectral density *P*(*ω*) of *ν*_*N*_(*t*)

In a network of a finite number *N* of neurons, the instantaneous firing rate *ν*_*N*_(*t*) displays endogenous fluctuations. Indeed, due to the finite number of spikes emitted by such a network in relatively small time intervals, the counting Poissonian statistics makes *ν*_*N*_(*t*) well described by a stochastic variable whose variance scales as 1/*N* [15, 27, 41]. These activity fluctuations can be seen as an ongoing stimulation of the infinite-size network and can be obtained by introducing an effective stochastic driving force different for each eigenmode of the FP operator [15]. Following this approach and generalizing Eq (12) to the case of a finite-size network, we obtain the following stochastic firing-rate equations

$$\begin{array}{c} \left\{ \begin{array}{c} {\dot{\vec{a}}}_{0}=(\Lambda_{0}+W_{0}\;{\dot{\mu }}_{y})\;{\vec{a}}_{0}+{\vec{w}}_{0}\;{\dot{\mu }}_{y}+{\vec{\psi }}_{0}\;\eta_{N}\\ {\dot{\mu }}_{y}=(\mu_{I}\left(\nu_{N}\right)-\mu_{y})/\tau_{s}\\ \nu =\Phi_{0}+{\vec{f}}_{0}\cdot {\vec{a}}_{0}\\ \nu_{N}=\nu +\eta_{N} \end{array}\;,\right. \end{array}$$ (18)

where *η*_*N*_(*t*) models the finite-size fluctuations of the instantaneous firing rate *ν*(*t*) in the infinite-size limit. For large enough *N*, *η*_*N*_ is well approximated by a Gaussian memoryless white noise, with zero mean and variance *ν*(*t*)/*N*. In the above equations the coefficients depend on *ν*_*N*_(*t*) instead of *ν*(*t*), as the infinitesimal mean *μ*_*I*_ and variance $\sigma_{I}^{2}$ are functions of the instantaneous firing rate of the presynaptic neurons. The additional coefficients $\vec{\psi_{0}}=\left\{\psi_{n}\left(v_{\text{res}},y\right){|}_{y=\mu_{y}}\right\}$ result from having incorporated finite-size fluctuations to the boundary condition on the flux conservation of the realizations *V*(*t*) exiting from *v*_thr_ and reentering in *v*_res_ [15].

In the *N* → ∞ limit, the network dynamics can be set into an asynchronous state such that *ν*(*t*) = ν* is an equilibrium point [ν* = Φ_0_(ν*)] with local stability [$\Phi_{0}^{\prime }\left(\nu^{*}\right)<1$] and the single-neuron spiking activity is asynchronous [22, 25]. In this state, finite-size fluctuations bring *ν*_*N*_(*t*) to wander around ν*, and Eq (18) can be linearized by neglecting the terms of order higher than $O(\eta_{N})$ in the Taylor’s series expansion around *ν*(*t*) = ν*. From this linearized dynamics, the Fourier transform *ν*_*N*_(*ω*) can be obtained (see [15, 32] for details), and the power spectral density *P*(*ω*) = |*ν*_*N*_(*ω*)|^2^ turns out to be

$$\begin{array}{c} P\left(\omega \right)=\frac{1+2\;\text{Re}[{\vec{f}}_{0}\cdot {\left(i\omega I-\Lambda_{0}\right)}^{-1}{\vec{\psi }}_{0}]}{{\left|1-[\Phi_{0}^{\prime }+i\omega {\vec{f}}_{0}\cdot {\left(i\omega I-\Lambda_{0}\right)}^{-1}{\vec{w}}_{0}]r\left(i\omega \right)\right|}^{2}}\frac{\nu^{*}}{N}\;, \end{array}$$ (19)

which is the expression detailed in Eq (17).

### Identification of mean-field parameters

To find the parameters for the numerical simulations (with NEST [30] and the high-performance custom simulator implementing the event-based approach described in [31]), the following procedure has been adopted.

A firing rate equilibrium point ν* was fixed a priori, and the contour line in the (*μ*, *σ*^2^) plane defined by Φ(*μ*, *σ*^2^) = ν* was numerically determined. Φ(*μ*, *σ*^2^) was computed analytically for the VIF [21] and LIF [25, 33, 34] neuron models. For the EIF neuron model, in the absence of an analytical expression for Φ(*μ*, *σ*^2^) valid in all the considered conditions, we used a numerical cubic interpolation (using the Matlab – The MathWorks, Natick, MA – function interp2) passing through samples obtained from NEST simulation data. Depending on the regime of interest (noise- or drift-dominated), a proper point was chosen along the iso-frequency line Φ(*μ*, *σ*^2^) = ν*.

Once determined *μ* and *σ*^2^, also *J* can be determined by imposing the value of $\frac{d}{d\nu }\Phi \left(\mu ,\sigma^{2}\right)$ at the equilibrium point ν*, which is directly related to its degree of stability [15]. Indeed, by taking into account Eq (2), it is sufficient to solve the following second-order equation in *J*:

$$\begin{array}{c} \frac{d}{d\nu }\Phi \left(\mu ,\sigma^{2}\right)=\tau_{m}CJ(\frac{\partial \Phi }{\partial \mu }+J\frac{\partial \Phi }{\partial \sigma^{2}}) \end{array}$$ (20)

where both ∂Φ/∂*μ* and ∂Φ/∂*σ*^2^ can be suitably computed from Φ(*μ*, *σ*). Among the possible solutions, we take the one corresponding to the stable equilibrium point Φ(*μ*, *σ*^2^) = ν* with the highest firing rate, since it is related to the proper range of *ν* values.

The final step is to determine mean *μ*_*ext*_ and variance $\sigma_{ext}^{2}$ of the external current *I*_*ext*_ to be added to the recurrent synaptic contribution in order to obtain the chosen *μ* and *σ*^2^. *I*_*ext*_ is the sum of a constant current bias *I*_*DC*_ and a Poissonian spike train produced by *C*_*ext*_ independent sources firing at rate *ν*_*ext*_ through an instantaneous synaptic transmission with efficacy *J*_*ext*_. Under diffusion approximation, *I*_*ext*_ is a memoryless Wiener process and by imposing the spike rate *C*_*ext*_ *ν*_*ext*_ from the external neurons, the two remaining parameters *I*_*DC*_ and *J*_*ext*_ are uniquely determined, in the distribution of delays case, by solving the following system

$$\begin{array}{c} \left\{ \begin{array}{c} \mu =\tau_{m}JC\nu^{*}+\tau_{m}J_{ext}C_{ext}\nu_{ext}+R\;I_{DC}\\ \sigma^{2}=\tau_{m}J^{2}C\nu^{*}+\tau_{m}J_{ext}^{2}C_{ext}\nu_{ext} \end{array}\;.\right. \end{array}$$ (21)

When the non-instantaneous synaptic transmission is incorporated, the recurrent synaptic efficacy has to be simply rescaled by the time constant *τ*_s_, i.e., *J* → *J*/*τ*_s_. This is the approach used to design all the VIF, LIF and EIF neuron networks analyzed in Figs 5–7.

The multi-modular networks devised to obtain the results shown in Figs 8–10 are composed of *N*_*E*_ excitatory and *N*_*I*_ inhibitory LIF neurons sparsely connected, each receiving synaptic inputs from *C*_*E*,*ext*_ and *C*_*I*,*ext*_, respectively, external Poisson-like excitatory neurons firing at rate *ν*_*ext*_ = 10 Hz. Neurons in the networks do not receive any additional current bias (*I*_*DC*_ = 0). As for the single-module networks, synapses with external neurons instantaneously transmit spikes, and their efficacies *J*_*ext*_ lead all neurons to fire at rates *ν*_*E*_ = *ν*_*I*_ = ν* = 10 Hz in the absence of recurrent synapses (uncoupled network). The other connectivity parameters are chosen starting from this condition, firstly by setting up an average number *C*_*XI*_ (with *X* ∈ {*E*, *I*}) of recurrent inhibitory connections, each with the same synaptic efficacy *J*_*XI*_. The value of *J*_*XI*_ is chosen to explore the three regimes shown in Fig 8 when *c*_*XI*_ = *C*_*XI*_/*N*_*I*_ varies from 0 to 100%. The external coupling strength *J*_*ext*_ is increased according to the current value of *C*_*XI*_ to keep unchanged the rate ν* = 10 Hz. Finally, *C*_*EE*_ = *C*_*IE*_ recurrent connections from excitatory neurons are randomly chosen, by replacing the same number of synaptic contacts with external neurons. This operation does not change the equilibrium properties, provided that excitatory recurrent and external synapses have the same efficacy: *J*_*EE*_ = *J*_*IE*_ = *J*_*ext*_.

Following this design strategy, all the E-I networks share the same configuration with only one exception. In Fig 10, instead of varying *C*_*XI*_, we kept it small and fixed (*c*_*XI*_ = 1%), and we replaced a variable fraction of the external excitatory synapses *C*_*I*,*ext*_ directed to the inhibitory neurons with the same number of contacts from randomly chosen recurrent excitatory neurons. This allowed to keep constant the total number *C*_*IE*_ + *C*_*I*,*ext*_ of excitatory synapses received by each inhibitory neuron.

### Details on the numerical simulations

The software used to obtain the results presented in this paper is available upon request. All the simulated IF neuron networks working in asynchronous state (Figs 5–7) are modeled by considering a sparse recurrent connectivity with connection probability *C*/*N*, being *C* the average number of synapses per neuron. Each neuron receives an external input from independent Poissonian spike trains with frequency *C*_*ext*_ *ν*_*ext*_ through instantaneous synapses with efficacy *J*_*ext*_. Spikes are always delivered to post-synaptic neurons with a minimum delay *δ*_*min*_. The values of both the synaptic time constant *τ*_s_ and the decay constant *τ*_d_ of the exponential delay distribution are 0, 1, 2, 4, 8, 16, 32, 64 ms. For LIF and EIF neurons, the stochastic current due to the Poissonian spiking activity of external neurons is incorporated as an equivalent (under the diffusion approximation) Wiener (Gaussian) process with mean *μ*_*ext*_ and variance $\sigma_{ext}^{2}$

$$\begin{array}{c} \left\{ \begin{array}{c} \mu_{ext}=C_{m}\;J_{ext}\;C_{ext}\;\nu_{ext}\;10^{-3}\\ \sigma_{ext}^{2}={\left(C_{m}\;J_{ext}\right)}^{2}C_{ext}\;\nu_{ext}\;10^{-3}/dt \end{array}\;,\right. \end{array}$$ (22)

where, in order to have the moments of the current expressed in pA, the firing rate *ν*_*ext*_ (usually expressed in Hz) is rescaled by one thousand to have it in ms^−1^.

The VIF neuron networks in Figs 5 and 6 are composed of the VIF (‘VLSI’ integrate-and-fire) neurons introduced in [21]. This model is an extended version of the standard ‘perfect integrate-and-fire’ (PIF) neuron model introduced in [39]: in addition to a constant leakage current *f*(*V*) = *β*, which here we consider as a part of the current bias *I*_*DC*_, a reflecting barrier at *V* = 0 is set to avoid a divergent diffusion towards negative membrane potentials. Contrary to the PIF neuron, this makes the VIF neuron capable of having a non-zero (positive) mean firing rate also under subthreshold regimes, i.e., for negative drifts (*μ* < 0). For the VIF neuron, input-output gain function Φ(*μ*, *σ*), eigenfunctions and eigenvalues of the related FP operator have explicit analytical expressions [15, 21, 32], which make this model particularly suited for matching theory and simulations. The parameters for the VIF neuron networks used in this paper are listed in Table 1. For this model the natural unit of measure for the membrane voltage is the firing threshold *v*_thr_.

**Table 1:** Parameters of the VIF neuron networks at drift-dominated (DD) and noise-dominated (ND) regimes (Figs 5 and 6).

Parameter	Value (DD)	Value (ND)
Number of excitatory neurons *N*	2000	2000
Mean excitatory synapses per neuron *C*	100	100
Excitatory PSP amplitude due to recurrent spikes *J* [*v*_thr_]	0.0075	0.014
Minimum transmission delay *δ*_*min*_ [ms]	10	10
External spike rate *C*_*ext*_ *ν*_*ext*_ [Hz]	3000	3000
Excitatory PSP amplitude due to external spikes *J*_*ext*_ [*v*_thr_]	0.0114	0.0726
Current bias *I*_*DC*_ [*v*_thr_/ms]	−0.032	−0.242
Refractory period *τ*_*ref*_ [ms]	0	0
Firing threshold *v*_thr_	1	1
Resting potential *V*_*E*_ [*v*_thr_]	0	0
Reset potential *v*_res_ [*v*_thr_]	0	0
Firing rate ν* [Hz]	10	10

For the LIF [18] and the EIF [42] neuron networks corresponding to the results shown in Fig 7, the parameters at drift- (DD) and noise-dominated (ND) regimes are listed in Tables 2 and 3, respectively.

**Table 2:** Parameters of the simulated LIF neuron networks at drift-dominated (DD) and noise-dominated (ND) regimes (Figs 7 and 4).

Parameter	Value (DD)	Value (ND)
Number of excitatory neurons *N*	2000	2000
Mean excitatory synapses per neuron *C*	100	100
Excitatory PSP amplitude due to recurrent spikes *J* [mV]	0.140	0.213
Minimum transmission delay *δ*_*min*_ [ms]	3	3
External spike rate *C*_*ext*_ *ν*_*ext*_ [Hz]	12000	12000
Excitatory PSP amplitude due to external spikes *J*_*ext*_ [mV]	0.272	1.518
Current bias *I*_*DC*_ [nA]	−1.23	−9.16
Membrane time constant *τ*_m_ [ms]	20	20
Refractory period *τ*_*ref*_ [ms]	0	0
Firing threshold *v*_thr_ [mV]	20	20
Membrane capacitance *C*_*m*_ [pF]	500	500
Resting potential *V*_*E*_ [mV]	0	0
Reset potential *v*_res_ [mV]	0	0
Firing rate ν* [Hz]	40	40

**Table 3:** Parameters of the EIF neuron networks at drift-dominated (DD) and noise-dominated (ND) regimes (Fig 7).

Parameter	Value (DD)	Value (ND)
Number of excitatory neurons *N*	2000	2000
Mean excitatory synapses per neuron *C*	100	100
Minimum transmission delay *δ*_*min*_ [ms]	3	3
External spike rate *C*_*ext*_ *ν*_*ext*_ [Hz]	12000	12000
Excitatory PSP amplitude due to external spikes *J*_*ext*_ [mV]	0.0804	1.76
Spike rate from external neurons *C*_*ext*_ [Hz]	12000	12000
Current bias *I*_*DC*_ [pA]	−8.46	−223
Membrane time constant *τ*_m_ [ms]	10	10
Refractory period *τ*_*ref*_ [ms]	1.7	1.7
Spike slope factor Δ_*T*_ [mV]	3.48	3.48
Firing threshold *v*_thr_ [mV]	−59.9	−59.9
Membrane capacitance *C*_*m*_ [pF]	500	500
Resting potential *V*_*m*_ [mV]	−65	−65
Leak potential *E*_*L*_ [mV]	−65	−65
Reset potential *v*_res_ [mV]	−68	−68
Leak conductance *g*_*L*_ = *C*_m_/*τ*_m_ [nS]	50	50
Excitatory PSP amplitude due to recurrent spikes *J* [mV]	0.134	0.272
*V*_*peak*_ [mV]	0	0
Firing rate ν* [Hz]	40	40

In Fig 3A and 3B we simulated a set of 4000 uncoupled LIF neurons with same single-neuron parameters as in Fig 7A. Each neuron received an independent source of Poissonian spikes with rate *Cν* ∈ [8.06, 205] kHz (Fig 3A) and [0.504, 12.8] kHz (Fig 3B), and with synaptic efficacy *J* = 0.0114 mV and 0.183 mV in Fig 3A and 3B, respectively. In both cases *I*_ext_ = −46.1 pA and *ν*_ext_ = 0 for *σ*_ext_ = 0. For *σ*_ext_ = 0.5 mV and 2.0 mV, *I*_ext_ = −50.2 pA and −112 *pA*, and the added white noise current had moments set as in Eq (22) with *C*_ext_ *ν*_ext_ = 5.42 Hz and 86.8 Hz, respectively. In both cases, *σ*_ext_ > 0 and *J*_ext_ = 1.52 mV. In Fig 3C, a network of excitatory LIF neurons was simulated by taking the same parameters as in Panel B with *σ*_ext_ = 2.0 mV, with two exceptions: the independent source of Poissonian spikes has rate *Cν* ∈ [0.505, 10.1] kHz and that each neuron receives 50 synapses from other cells in the network. In all panels, for each input *Cν*, simulations were 2 seconds long, and asymptotic firing rates were estimated discarding the first second to avoid transient effects. To estimate the mean and SEM of the output firing rates, 10 independent simulations were performed.

Table 4 lists the parameters for the multi-modular E-I networks shown in Figs 8–10, with average numbers of external connections *C*_*E*,*ext*_ = *C*_*I*,*ext*_ = 1800, firing rate *ν*_*ext*_ = 10 Hz, percentage of excitatory connections *c*_*EE*_ = *c*_*IE*_ = 5%, and recurrent inhibitory synaptic efficacies *J*_*EI*_ = *J*_*II*_ = −0.05 mV. Figs 8 and 9 have been obtained by changing *c*_*XI*_ and the excitatory synaptic efficacies (expressed in mV) as

$$\begin{array}{c} J_{ext}=J_{EE}=J_{IE}=\left(9.56\times 10^{-5}\;c_{XI}+0.0234\right)c_{XI}+0.0485\;. \end{array}$$

**Table 4:** Parameters of the LIF neuron networks used for the multi-modular tests (Figs 8, 9 and 10).

Parameter	Value
Number of inhibitory neurons *N*_*inh*_	1000
Number of excitatory neurons *N*_*exc*_	4000
Membrane time constant *τ*_m_ [ms]	20
Refractory period *τ*_*ref*_ [ms]	0
Firing threshold *v*_thr_ [mV]	20
Membrane capacitance *C*_*m*_ [pF]	100
Resting potential *E*_*L*_ [mV]	0
Reset potential *v*_res_ [mV]	10
Time step *dt* [ms]	0.05
Minimum delay *δ*_*min*_ [ms]	3
Simulation time [ms]	61000

Fig 10 has been obtained by setting synaptic efficacies *J*_*ext*_ = *J*_*EE*_ = *J*_*IE*_ = 0.0487 mV, *J*_*II*_ = *J*_*EI*_ = −0.05 mV, connections with external neurons *C*_*E*,*ext*_ = *C*_*I*,*ext*_ = 1960, and recurrent connection probability 0.01% for all neurons.

The simulations performed with NEST have been implemented using iaf_psc_delta and iaf_psc_exp neuron models. The recurrent connections were introduced following the fixed_indegree rule to lower quenched noise effects, relying on the static_synapse model. In the case of distribution of delays and instantaneous synaptic transmission the NEST neuronal model adopted was iaf_psc_delta. The distribution of delays was exponential_clipped from the NEST library, where the following parameters were used: lambda = 1/*τ*_s_, low = *δ*_*min*_ + *dt*, high = float(‘inf’), with *dt* as integration time step. Synaptic efficacies expressed in mV were converted in pC (total electric charge) by multiplying them by *C*_*m*_.

## Supporting information

S1 Appendix Theoretical derivation and cascade of synaptic filters. Details about the dimensional reduction of the firing rate dynamics and about networks of neurons incorporating simultaneously both non-instantaneous synaptic transmission and heterogeneous propagation delays.
(PDF)
