# Supplementary material for: Dimensional reduction in networks of non-Markovian spiking neurons: Equivalence of synaptic filtering and heterogeneous propagation delays
Source: PLoS Comput Biol. 2019 Oct 8;15(10):e1007404. doi: 10.1371/journal.pcbi.1007404 (PMC6799936; doi:10.1371/journal.pcbi.1007404)
Supplement: S1 Appendix — Details about the dimensional reduction of the firing rate dynamics and about networks of neurons incorporating simultaneously both non-instantaneous synaptic transmission and heterogeneous propagation delays. (PDF) [file pcbi.1007404.s001.pdf]

Dimensional reduction in networks of non-Markovian spiking neurons: equivalence of synaptic filtering and heterogeneous propagation delays

Maurizio Mattia[1*], Matteo Biggio[2], Andrea Galluzzi[1], Marco Storace[2]

1 Istituto Superiore di Sanità, 00161 Roma, Italy
2 DITEN, University of Genoa, 16145 Genova, Italy

* maurizio.mattia@iss.it

Appendix

Dimensional reduction of the firing rate dynamics

Here we detail how to obtain the dimensional reduction of the firing rate dynamics described in the *Theoretical results* section.

By combining Eqs. (7) and (8), the total firing rate can be then rewritten as

$$\begin{aligned}\nu &= \int_{-\infty}^{\infty} \nu_y(y,t)\,dy \\ &= \Phi(\mu_y,\nu)\int_{-\infty}^{\infty}\rho_y(y,t)\,dy + \\ &\quad \sum_{n\neq 0} f_n(\mu_y,\nu)\int_{-\infty}^{\infty} a_n(y,t)\,dy + \mathcal{O}(\sigma_I) \\ &\equiv \Phi_0(\nu) + \sum_{n\neq 0} a_{0n}\, f_{0n}(\nu) + \mathcal{O}(\sigma_I)\,,\end{aligned} \tag{S1}$$

where the 0-th order terms of the Taylor expansions are defined as $\Phi_0(\nu) \equiv \Phi(\mu_y,\nu)$ and $f_{0n}(\nu) \equiv f_n(\mu_y,\nu)$, together with the introduction of the new integral expansion coefficients $a_{0n}(t) \equiv \int_{-\infty}^{\infty} a_n(y,t)\,dy$. The next step to find a self-consistent approximated spectral expansion of Eq. (3) is to work out the dynamics of these $a_{0n}(t)$. This can be done by integrating both sides of Eq. (5) and constraining the additional diffusion term in $\mathcal{L}_{xy}$ to be as small as the fluctuation size of y: $\sigma^2(y) = \mathcal{O}(\sigma_I^2)$. The dynamics of the integral expansion coefficients reduces to

$$\begin{aligned}\dot{a}_{0n} &= \int_{-\infty}^{\infty}\lambda_n(y)\,a_n(y,t)\,dy + \\ &\quad \sum_q \int_{-\infty}^{\infty}\langle\psi_n|\partial_y(\frac{y-\mu_I}{\tau_s}a_q\phi_q)\rangle\,dy + \mathcal{O}(\sigma_I^2) \\ &= \lambda_{0n}\,a_{0n} - \frac{\mu_y-\mu_I}{\tau_s}\sum_q \langle\partial_y\psi_n|\phi_q\rangle|_{y=\mu_y}a_{0q} + \mathcal{O}(\sigma_I)\,,\end{aligned}$$

where we integrated by parts the second integral and set $\lambda_{0n} \equiv \lambda_n(\mu_y)$. Note that, after this simplification the neglected terms are $\mathcal{O}(\sigma_I)$ due to the part of the Taylor's expansion we are not taking into account, and whose contribution is larger than the aforementioned terms related to $\sigma^2(y)$. This equation for a_{0n} can be further recast as follows, by taking into account Eq. (9):

$$\dot{a}_{0n} = \lambda_{0n}\,a_{0n} + \dot{\mu}_y\sum_q\langle\partial_y\psi_n|\phi_q\rangle|_{y=\mu_y}a_{0q} + \mathcal{O}(\sigma_I)\,. \tag{S2}$$

Cascade of synaptic filters and delay distributions

Biological networks of neurons incorporate simultaneously both non-instantaneous synaptic transmission and heterogeneous propagation delays. In this paper, we studied and compared these two transmission mechanisms one at a time. However, Eq. (12) can be extended to include both synaptic filters and delay distributions. Indeed, the spike rate $\tilde{\nu}(t)$ each neuron receives at time t is given by the convolution of $\nu(t)$ and the distribution $\rho_d(\delta)$ of delays δ as shown in Sec. "Equivalence of non-instantaneous synaptic transmission and distribution of axonal delays". This affects the instantaneous mean current μ_y and leads to generalize the firing rate equation as follows:

$$\begin{cases} \dot{\vec{a}}_0 = (\mathbf{\Lambda}_0 + \mathbf{W}_0\,\dot{\mu}_y)\,\vec{a}_0 + \vec{w}_0\,\dot{\mu}_y \\ \dot{\mu}_y = (\mu_I(\tilde{\nu}) - \mu_y)\,/\tau_s \\ \dot{\tilde{\nu}} = (\nu - \tilde{\nu})\,/\tau_\mathrm{d} \\ \nu = \Phi_0 + \vec{f}_0 \cdot \vec{a}_0 \end{cases} . \tag{S3}$$

Similarly to what shown in the *Theoretical results* section, this dynamics is equivalent to the one of a network with instantaneous synaptic transmission – Eq. (14) – with a distribution of delays $\rho_d(\delta)$ implementing a second-order low-pass filter:

$$\rho_d(\delta) = \beta(\delta|\tau_\mathrm{s}, \tau_\mathrm{d}) \equiv (e^{-\delta/\tau_\mathrm{d}} - e^{-\delta/\tau_\mathrm{s}})/(\tau_\mathrm{d} - \tau_\mathrm{s})\,, \tag{S4}$$

which for $\tau_\mathrm{d} = \tau_\mathrm{s}$ reduces to

$$\rho_d(\delta) = \alpha(\delta|\tau_\mathrm{d}) \equiv \delta e^{-\delta/\tau_\mathrm{d}}/{\tau_\mathrm{d}}^2\,. \tag{S5}$$

Therefore, the combination of synaptic filtering and propagation delay distribution can be effectively described as a cascade of two first-order filters, leading to firing rate dynamics as for a network with only distribution of delays, further generalizing the descriptive power of the perturbative approach proposed in this paper. As shown in Fig. 11, this picture is confirmed by simulations of E-I LIF neuron networks. Indeed, the power spectra $P(\omega)$ from different network types display a remarkable agreement. As expected, the $P(\omega)$s of $\nu(t)$ are shaped differently if different combinations of transmission components are incorporated, even if the total time scale is preserved ($\tau_\mathrm{s} + \tau_\mathrm{d} = 16$ ms). Indeed, when only one component is considered (Fig. 11A), $\rho_d(\delta)$ is exponential (Eq. (15)) whereas, for the combined case, delay distribution have a unimodal shape with rise and decay as prescribed by Eq. (S4) (Fig. 11B) or by Eq. (S5) when $\tau_\mathrm{d} = \tau_\mathrm{s}$ (Fig. 11C).

We remark that synaptic filters and equivalent delay distributions are interchangeable (Fig. 11B-C). For instance, slow synaptic filters with $\tau_\mathrm{s} = 12$ ms and narrow delay distributions with $\tau_\mathrm{d} = 4$ ms give rise to the same power spectra as those measured in networks with reversed features of the transmission components ($\tau_\mathrm{s} = 4$ ms and $\tau_\mathrm{d} = 12$ ms). Furthermore, if a rising-and-decaying post-synaptic current (α-PSC, a second-order low-pass filter as in [6] with rise time $\tau_r = \tau_\mathrm{s}$) is incorporated without delay distribution, the resulting $P(\omega)$ appears to overlap with the power spectral density of a network with $\rho_d(\delta) = \alpha(\delta|\tau_\mathrm{d})$, or equivalently, with a cascade of filters ($\tau_\mathrm{s} = \tau_\mathrm{d} = 8$ ms, Fig. 11C). The network setting including α-PSC is not described by the approximated dynamics we derived in Eq. (12). However, the observed overlap between spectra is a strong evidence of the capability of our perturbative approach to describe also Markovian neurons with dimension larger than two. Indeed, α-PSC can be described simply by adding a first-order differential equation to Eq. (1) [6].
